# Using nanopore sequencing to identify fungi from clinical samples with high phylogenetic resolution

**DOI:** 10.1038/s41598-023-37016-0

**Published:** 2023-06-16

**Authors:** Atsufumi Ohta, Kenichiro Nishi, Kiichi Hirota, Yoshiyuki Matsuo

**Affiliations:** 1 Department of Human Stress Response Science, Institute of Biomedical Science, Kansai Medical University, 2-5-1 Shin-machi, Hirakata, Osaka 573-1010 Japan; 2 Department of Anesthesiology and Intensive Care, Osaka Red Cross Hospital, Osaka, Japan

**Keywords:** Bioinformatics, Fungal genomics, Fungal infection, Microbiology techniques, DNA sequencing

## Abstract

The study of microbiota has been revolutionized by the development of DNA metabarcoding. This sequence-based approach enables the direct detection of microorganisms without the need for culture and isolation, which significantly reduces analysis time and offers more comprehensive taxonomic profiles across broad phylogenetic lineages. While there has been an accumulating number of researches on bacteria, molecular phylogenetic analysis of fungi still remains challenging due to the lack of standardized tools and the incompleteness of reference databases limiting the accurate and precise identification of fungal taxa. Here, we present a DNA metabarcoding workflow for characterizing fungal microbiota with high taxonomic resolution. This method involves amplifying longer stretches of ribosomal RNA operons and sequencing them using nanopore long-read sequencing technology. The resulting reads were error-polished to generate consensus sequences with 99.5–100% accuracy, which were then aligned against reference genome assemblies. The efficacy of this method was explored using a polymicrobial mock community and patient-derived specimens, demonstrating the marked potential of long-read sequencing combined with consensus calling for accurate taxonomic classification. Our approach offers a powerful tool for the rapid identification of pathogenic fungi and has the promise to significantly improve our understanding of the role of fungi in health and disease.

## Introduction

The development of DNA metabarcoding, a genetic method for specimen identification, has provided great improvements and innovations for analyzing microbiota from environmental and clinical sources^1,2^. This technique typically amplifies a target marker gene, and sequences the resulting amplicons to characterize the microbial species present in a sample. Sequencing-based approaches have revolutionized the study of microbiota, enabling rapid and accurate detection of microbes, from multispecies communities without the need for time-consuming culture and laboratory isolation. As a result, the DNA metabarcoding provides comprehensive taxonomic profiles with greater phylogenetic resolution^3,4^.

Amplicon sequencing utilizes a polymerase chain reaction (PCR) with primer pairs to amplify specific DNA markers across species. To achieve broad coverage of species, the DNA markers must be flanked by conserved primer binding sites while also being sufficiently variable to differentiate between taxa. In bacteria, 16S ribosomal RNA (rRNA) gene, a sequence of approximately 1500 base pairs (bp), has been extensively used for community profiling at reasonable taxonomic resolution^5^. The bacterial 16S rRNA gene consists of interspersed variable (V1–V9) and conserved sequences, which renders it a suitable genetic marker for bacterial metabarcoding. The DNA marker commonly used to identify fungal species is the internal transcribed spacer (ITS) region located between the small subunit (SSU) and large subunit (LSU) rRNA genes^6^. The ITS region consists of two loci, ITS1 and ITS2, separated by the 5.8S rRNA gene. To identify the taxonomy of a sample, either one or both subregions can be targeted for sequencing. The non-coding ITS region has had faster mutation rate than SSU and LSU rRNA coding regions, resulting in accumulated sequence variations that help discriminate different taxa^7^. Bacterial and fungal DNA markers are present in multiple copies of the genome, making them technically advantageous for amplicon sequencing^8–10^. Regions within the rRNA operon are readily targeted for PCR amplification, even with small amounts of template DNA. Thus, DNA metabarcoding targeting rRNA operons has been extensively employed as a standardized method for microbial identification^5,11^.

High-throughput sequencing platforms, which are also referred to as second-generation sequencing technologies, have made significant contributions to the advancement of sequence-based molecular taxonomy^12^. Approaches using high-throughput sequencing can generate accurate sequence data on a massive scale. However, their technical restrictions limit the read length, and the Illumina MiSeq, a prime example of a high-throughput sequencing platform, generates reads with a maximum of 600 bp (2 × 300 bp). Owing to this limitation, a partial sequence of the bacterial 16S rRNA gene (e.g., V1–V2 and V3–V4) and a sublocus of the fungal ITS region have been targeted for metabarcoding using the current high-throughput sequencing systems. In DNA metabarcoding, the amplicon length is a critical factor that determines the accuracy and precision of taxonomic identification^4^. Short amplicon sequences provide limited resolution for many bacterial and fungal taxa, which is insufficient for the species-level characterization of the microbiota^1,13,14^.

More recently, new sequencing platforms, such as those from Pacific Biosciences^15^ and Oxford Nanopore Technologies^16^, have emerged as a technological innovation. They offer remarkable advantages in overcoming the limitations of short-read sequencing. A nanopore sequencing technology generates long sequencing reads without a theoretical length limit^17^. DNA metabarcoding with long amplicons could potentially provide substantially improved phylogenetic resolution compared to short-read sequencing, resulting in more accurate taxonomic classification. Another competitive advantage of nanopore sequencing is that the sequencing data are produced in real time. Unlike conventional sequencing methods where data become available after the completion of the run, the nanopore platform enables rapid access to sequencing results. Real-time data streaming is particularly useful for time-critical analyses such as the rapid identification of pathogens for diagnostic purposes^18,19^. Given the features of nanopore sequencing, we previously established a method for DNA metabarcoding that targets the full-length 16S rRNA gene^20^. This workflow has proven useful for the rapid identification of bacterial pathogens and human microbiome profiling with improved taxonomic resolution^21,22^.

In the present study, we applied our nanopore long-read sequencing methodology to the molecular identification of fungi. We present a DNA metabarcoding workflow that includes DNA extraction from clinical samples, PCR amplification, and long-read sequencing on a nanopore MinION device. We also provided bioinformatics pipelines to generate highly accurate consensus sequences from nanopore reads and align them against fungal reference genome assemblies for taxonomic assignment. Long amplicons spanning ITS–LSU regions of different lengths were tested to evaluate taxonomic resolution, and the feasibility of the approach in a clinical context was explored using a specimen derived from a patient diagnosed with pneumonia.

## Results

### Selecting primer sets for fungal metabarcoding

**Figure 1:**
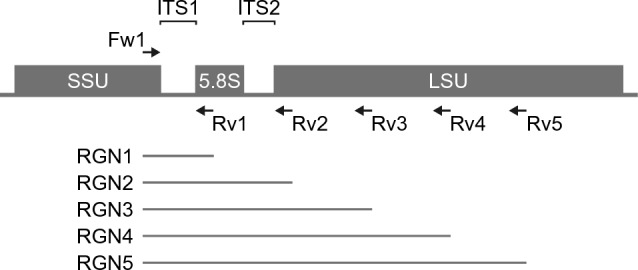
Amplicon sequencing targeting the fungal rRNA operon. The locations of primer sites (Fw1, a shared forward primer; Rv1–5, reverse primers) and target regions (RGN1–5) are indicated. ITS, internal transcribed spacer; SSU, small subunit; LSU, large subunit.

The use of longer sequences can significantly enhance the amount of genetic information, improving the resolution for phylogenetic analysis. Here, we employed the nanopore long-read sequencing technology to target genomic DNA sequences spanning the ITS region and the LSU rRNA gene within the fungal rRNA operon for taxonomic identification through sequence analysis. To obtain amplicons spanning ITS–LSU regions of different lengths (RGN1–5), we selected five universal primer sets, including one shared forward primer (ITS1-F_KYO2) and five distinct reverse primers (ITS2-KYO2, ITS4, LR5, LR7, and RCA95m), which have been established in previous studies (Fig. 1 and Table 1)^23–27^. Primer binding sites were searched in the reference genome sequences of selected 11 fungal species with a broad phylogenetic range (Supplementary Data S1 and S2; Supplementary Tables S1 and S2). The amplicon lengths (excluding the barcodes and nanopore anchors) were estimated for each species with the following average values (bases): 323.8 for RGN1, 675.8 for RGN2, 1586.7 for RGN3, 2070.8 for RGN4, and 2758.4 for RGN5 (Supplementary Table S3). All amplicon sizes except for RGN1 exceeded the maximum read length that can be supported by high-throughput short-read sequencing technology, demonstrating the practicality of long-read sequencing.

**Table 1:** PCR primers used to amplify fungal rRNA gene.

Primer	Original name	Sequence (5′–3′)		References
		Nanopore anchor	Region-specific sequence	
Fw1	ITS1-F_KYO2^a^	TTTCTGTTGGTGCTGATATTGC	TAGAGGAASTAAAAGTCGTAA	^23^
Rv1	ITS2_KYO2	ACTTGCCTGTCGCTCTATCTTC	TTYRCTRCGTTCTTCATC	^23^
Rv2	ITS4	ACTTGCCTGTCGCTCTATCTTC	TCCTCCGCTTATTGATATGC	^24^
Rv3	LR5	ACTTGCCTGTCGCTCTATCTTC	TCCTGAGGGAAACTTCG	^25^
Rv4	LR7	ACTTGCCTGTCGCTCTATCTTC	TACTACCACCAAGATCT	^26^
Rv5	RCA95m	ACTTGCCTGTCGCTCTATCTTC	CTATGTTTTAATTAGACAGTCAG	^27^

^a^The sequence of the original ITS1-F_KYO2 was slightly modified to include degenerate bases.

### Generating highly accurate consensus sequences from nanopore sequencing data

**Figure 2:**
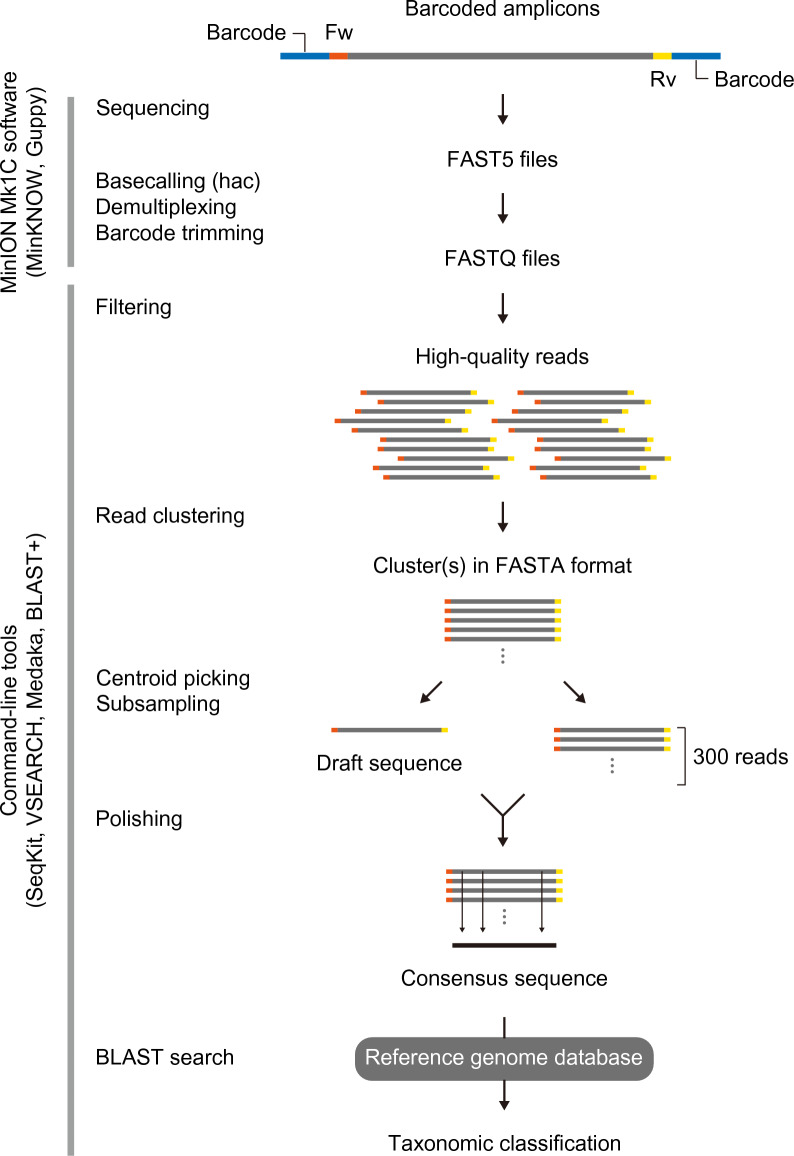
Overview of DNA metabarcoding workflow. The target gene marker is amplified using PCR with sample-specific barcodes and analyzed by nanopore sequencing. After demultiplexing, the reads are quality and size filtered, clustered, and polished for consensus calling. The resulting highly accurate consensus sequences are aligned against the reference genomes for taxonomic assignment. Fw, forward primer; Rv, reverse primer; hac, high accuracy.

The specificity and efficiency of amplification using these primer sets were validated by PCR using the genomic DNA isolated from *Aspergillus niger*. Primers were synthesized with 5' tails of 22 bases required for subsequent sequencing adapter attachment (Table 1). The original ITS1-F_KYO2 forward primer was developed for use with ITS2-KYO2 and ITS4, targeting the ITS1 sublocus and the entire ITS region^23^. The tailed ITS1-F_KYO2 primer (Fw1) was compatible with all five reverse primers (Rv1–5) used for PCR amplification, generating bands of the expected size for gel electrophoresis. We generated barcoded sequencing libraries and used the MinION nanopore sequencer to sequence them. To enhance sequence accuracy, we employed a consensus sequence generation approach to correct errors in the nanopore sequencing data (Fig. 2)^28^. The reads were filtered based on the average quality score and size thresholds defined for each target region. The resulting high-quality reads were clustered at 85–89% sequence similarity, and for all target regions, the majority of reads (> 99.5%) derived from *Aspergillus niger* isolates were successfully grouped into a single cluster (Table 2).

**Table 2:** Sequencing read statistics for *Aspergillus niger* sample.

Amplicon	Filtered reads			Primary cluster	
	Number of reads	Average length	Q-score	Number of reads	%^a^
RGN1	10,996	296.1	16.2	10,948	99.56
RGN2	12,472	621.9	15.9	12,468	99.97
RGN3	22,588	1521.1	15.6	22,588	100
RGN4	12,166	2006.8	15.8	12,151	99.88
RGN5	33,893	2682.1	15.7	33,856	99.89

^a^Percentage of reads in the primary cluster with the highest read count.

To determine the optimal subread coverage for consensus sequence generation, we tested five different read depths (30, 100, 300, 500, and 1000) for read polishing. A representative sequence (centroid) was selected from the cluster and used it to generate an error-corrected consensus sequence by polishing it with different numbers of sub-reads. At each position, the most frequent base was chosen to generate the consensus sequence. The sequence accuracy was assessed by aligning the consensus sequence generated at a given subread coverage with the reference genome of *Aspergillus niger* (GCF_000002855.3) (Supplementary Data S3). In most datasets, the lowest error rate was consistently observed at a depth of 300 reads, achieving 99.9–100% sequence accuracy (Supplementary Table S4); no beneficial effects were observed when the read depth was increased to 500 or more. Based on these observations, we set the subread coverage for consensus calling to 300 reads for subsequent analyses.

### Evaluating taxonomic resolution of the target rRNA gene regions for identifying *Aspergillus* species

We investigated the impact of target read length on taxonomic classification accuracy by performing a Basic Local Alignment Search Tool (BLAST)^29^ search against the RefSeq database, which contains 453 fungal genomes (Supplementary Table S5a). We used a reference amplicon sequence for *Aspergillus niger* as the query. For all the target regions, *Aspergillus niger* was correctly identified with the highest bit score (Supplementary Data S4). To validate the taxonomic assignment, we compared the bit score difference between the top hit (*Aspergillus niger*) and second-best hit(s) (non-target taxa) for each sequencing region. Given that the bit score difference is not affected by the length of the query sequence, it represents a useful metric for accurate read assignment^30^. When using RGN1 (ITS1 sublocus) or RGN2 (entire ITS region) as a query sequence in the BLAST search, three non-target *Aspergillus* species were ranked second, with a bit score difference of less than 20 and only two or three mismatches with *Aspergillus niger* (Table 3). Extending the target length to RGN3, including part of the LSU region, resulted in an increased difference in the bit score from the best hit, indicating a better resolution in the taxonomic classification. There was no considerable difference in the discriminatory power between RGN3, RGN4, and RGN5, suggesting that the LSU region downstream of the Rv3/LR5 primer site was not very informative for identifying *Aspergillus niger*.

**Table 3:** Comparison of BLAST results using five target regions for identifying *Aspergillus niger*.

Amplicon (bases^a^)	BLAST hits				Number of			Identity (%)
	Rank	Taxon	Accession	Diff^b^	Identical matches	Mis-matches	Gap opens	
RGN1 (257)	1	*A. niger*	NT_166520.1	–	257	0	0	100
	2	*A. eucalypticola*	NW_020290355.1	11	255	2	0	99.22
		*A. luchuensis*	NC_054852.1	11	255	2	0	99.22
		*A. tubingensis*	NW_023336289.1	11	255	2	0	99.22
RGN2 (586)	1	*A. niger*	NT_166520.1	–	586	0	0	100
	2	*A. eucalypticola*	NW_020290355.1	17	583	3	0	99.49
		*A. luchuensis*	NC_054852.1	17	583	3	0	99.49
		*A. tubingensis*	NW_023336289.1	17	583	3	0	99.49
RGN3 (1494)	1	*A. niger*	NT_166520.1	–	1494	0	0	100
	2	*A. luchuensis*	NC_054852.1	45	1486	8	0	99.47
		*A. tubingensis*	NW_023336289.1	45	1486	8	0	99.47
RGN4 (1979)	1	*A. niger*	NT_166520.1	–	1979	0	0	100
	2	*A. luchuensis*	NC_054852.1	44	1971	8	0	99.60
		*A. tubingensis*	NW_023336289.1	44	1971	8	0	99.60
RGN5 (2655)	1	*A. niger*	NT_166520.1	–	2655	0	0	100
	2	*A. luchuensis*	NC_054852.1	49	2646	9	0	99.66

^a^Length of the query reference sequence excluding the primers.

^b^Bit score difference between the top and second-best hits.

### Analyzing mixed communities of fungi

We applied our nanopore amplicon sequencing method to community profiling of fungi. The five target regions of the rRNA operon were amplified by PCR using a mixture of genomic DNA from 10 medically relevant fungal species. The amplicons were sequenced and the reads that passed the filtering conditions were clustered based on sequence similarity. For all five target regions, most reads (> 96.7%) were clustered into ten major groups, with the remainder constituting clusters with low read counts (Table 4). The draft sequence was polished using 300 randomly subsampled reads, resulting in ten consensus sequences for each dataset. These consensus sequences were then aligned against a fungal reference database, including 453 RefSeq and 3894 GenBank genomes for taxonomic classification (Supplementary Table S5). Nanopore amplicon sequencing with error correction enabled the discrimination of the ten expected fungal taxa present in the mock community (Supplementary Data S5 and Supplementary Table S6). There were no significant differences in the Shannon index, indicating that the community diversity (alpha diversity) was similar with no evident PCR biases between the target regions (Supplementary Table S7).

**Table 4:** Sequencing read statistics for a mock community sample.

Amplicon	Filtered reads			Ten major clusters	
	Number of reads	Average length	Q-score	Number of reads	%^a^
RGN1	12,020	278.4	16.8	11,950	99.42
RGN2	12,420	617.8	16.1	12,171	98.00
RGN3	18,930	1515.6	15.8	18,308	96.71
RGN4	10,291	2024.2	16.0	10,136	98.49
RGN5	12,100	2707.5	15.9	11,658	96.35

^a^Percentage of reads constituting ten major clusters for each dataset.

**Figure 3:**
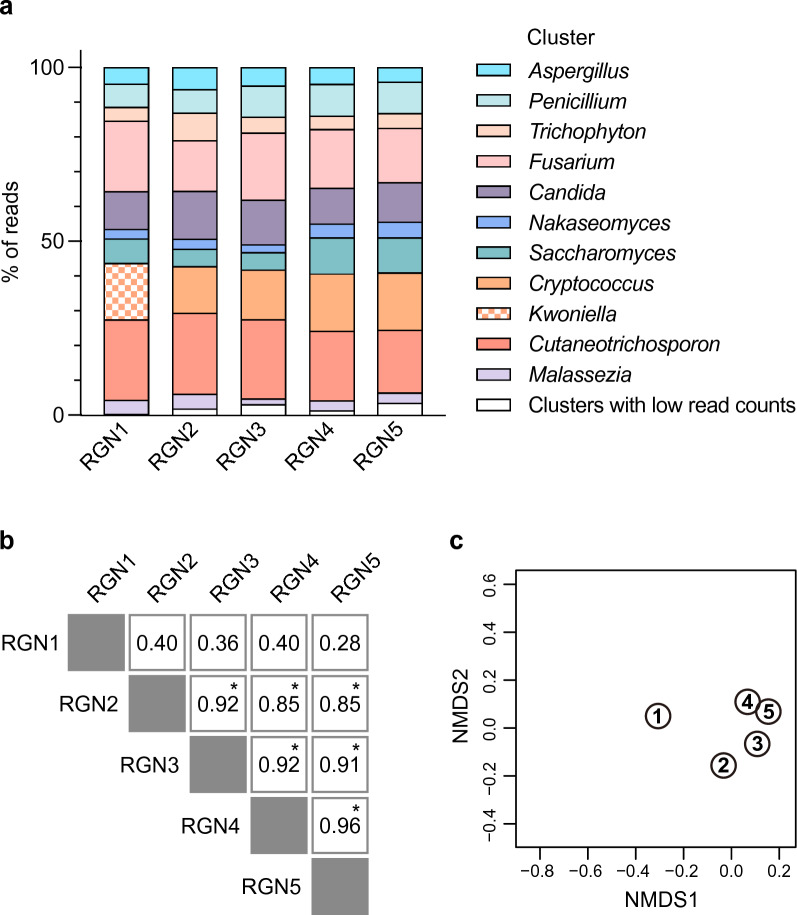
Taxonomic assignments for the fungal mock community standard using five sequencing regions. (**a**) Taxonomic composition of a 10-species fungal mock community was determined using nanopore amplification sequencing data. The community composition (genus level) was determined for each target region (RGN1–5) by BLASTN search against the reference genome using the consensus sequence as a query. The relative abundance of reads assigned to the expected target taxa is shown. (**b**) The Spearman’s rank correlation coefficients for pairwise comparison between the five sequencing regions. Asterisks indicate significant correlations at *P* < 0.05. (**c**) NMDS ordination based on Bray–Curtis dissimilarities among the five target regions. A closer distance between data points indicates a higher similarity in the fungal community composition. Numbers in the circles represent the target amplicons (RGN1–5).

Overall, each consensus sequence was correctly classified as a target taxon at the genus level (Fig. 3a), although the level of taxonomic resolution varied across different target regions (Supplementary Table S8). Amplicon length had no significant effect on the community composition, and the relative abundance of each taxon (cluster) was highly correlated and comparable across the amplified regions (Fig. 3b). The only exception was observed with the shortest RGN1 amplicon, where the consensus sequence generated from the cluster representing *Cryptococcus neoformans* was misassigned to a non-target species belonging to the neighboring genus *Kwoniella* with the highest bit score, followed by *Cryptococcus neoformans* ranked second in the BLAST analysis (Supplementary Table S8h). For RGN1, the reference sequence of *Kwoniella mangroviensis* differed from that of *Cryptococcus neoformans* by only one base, making it difficult to discriminate between these two fungal taxa. Differences in the community composition were also evaluated using the Bray–Curtis dissimilarity indices for each dataset (Supplementary Table S9). The non-metric multidimensional scaling (NMDS) plot illustrated a clear separation of the RGN1 sample from other experimental groups (Fig. 3c).

In comparison, the analyses with the other four regions (RGN2–5) exhibited a better resolution than RGN1, classifying eight of the ten taxa correctly to the species level with a sequence identity of 99.5% to 100% (Supplementary Table S8). Exceptions included *Trichophyton interdigitale*, which showed smaller query coverage in alignment with the reference genome and was placed at a lower rank in the BLAST analysis, regardless of the target amplicons (Supplementary Data S5). Instead, a closely related species *Trichophyton mentagrophytes* was assigned to the query in each dataset (Supplementary Table S8c). This may be attributed to the incomplete reference genome assembly in the searched database, where the rRNA gene locus of *Trichophyton interdigitale* split into two separate contigs (JAADCK010000556.1 and JAADCK010000559.1). *Trichophyton interdigitale* was excluded from subsequent analyses because of its incompatibility with the current database.

**Figure 4:**
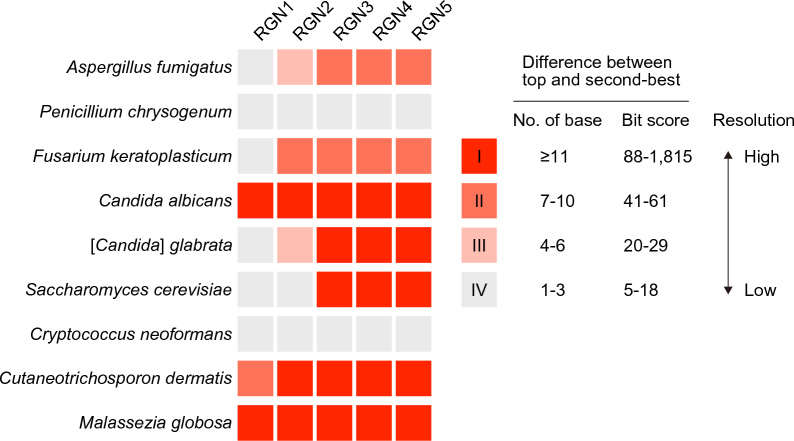
Comparison of taxonomic resolution for fungal species identification using different sequencing approaches. The classification results of each target region are categorized into four groups (I–IV) in accordance with bit score differences between the top and the second-best BLAST hits. The long-read sequencing approach shows superior discriminatory power, resulting in a more reliable and accurate taxonomic profiling.

We investigated the discriminatory power of each target region with respect to the accuracy of taxonomic assignment of individual fungal species. The bit score differences between the top BLAST hit and the second-best hits were calculated for each dataset, and the results were roughly classified into four categories (I–IV) in descending order of magnitude (Fig. 4). These were categorized according to the number of bases that differed between the top and second-best BLAST hits for each dataset. Category I, with a relatively large bit-score difference (≥ 88), represented the highest resolution. In this category, the sequence similarity between the top- and second-best hits was judged to be low, assigning the query to a specific taxon with a high probability of accuracy. When the results fell into category IV with a bit score difference of less than 20, there were only one to three base differences between the top and second-best hits, indicating that they shared almost identical sequences for the target regions that were difficult to discriminate from one another. RGN1 sequences were substantially conserved among members of each taxon for the six species in the mock community, indicating that RGN1 is ineffective for identifying fungal species. A higher resolution can generally be obtained by increasing the length of the target regions used for taxonomic classification. In fact, RGN3 provided better resolution than RGN1 and RGN2 and classified the query sequences to each of the given taxa with less uncertainty. Further extension of the amplicon length to RGN5 did not significantly improve the identification results. The exceptions included *Candida albicans* (Supplementary Table S8e), *Cutaneotrichosporon dermatis* (Supplementary Table S8i), and *Malassezia globosa* (Supplementary Table S8j). In identifying these species, RGN1 exhibited discriminatory power comparable to that of other longer target regions. In contrast, *Penicillium chrysogenum* (Supplementary Table S8b) and *Cryptococcus neoformans* (Supplementary Table S8h), even with the longest RGN5, were difficult to reliably differentiate from closely related species.

### Application to clinical specimens

Finally, we used a long-read amplicon sequencing approach to identify pathogenic fungi in a human clinical specimen. Sputum samples were collected from a patient with pneumonia and subjected to fungal culture tests to diagnose fungal infections. DNA was extracted from the sample and analyzed using nanopore amplicon sequencing for taxonomic identification. Phosphate-buffered saline solution (negative control), which was used as a diluent for sample collection, was processed in the same manner for all subsequent steps. Given that RGN1 provides less resolution and is often insufficient for correct taxonomic classification, longer target regions (RGN2–5) were used to identify fungal pathogens in human specimens. The four regions were successfully amplified from the experimental sample using PCR, generating amplicons of the expected size for each target. In contrast, the negative control samples produced no detectable bands on gel electrophoresis. We sequenced four experimental and negative control samples and filtered the reads to retain only those meeting size and quality criteria for subsequent analysis. Any reads from negative control samples did not pass filtering, precluding the risk of significant background contamination. Sequences obtained from the experimental samples were clustered for consensus calling. For each dataset, the majority of reads (97.6%) constituted a single cluster (Table 5), suggesting infection by a single fungal species. The consensus sequences generated from each target region were aligned against the fungal reference genome (RefSeq + GenBank) using the BLASTN program to report the taxonomy of the best hits (Supplementary Data S6). The query consensus sequences from each dataset were consistently assigned to *Candida albicans* with sequence identities of over 99.6% (Table 6). The reliability of these results was confirmed using a culture-based test to detect *Candida albicans*.

**Table 5:** Sequencing read statistics for a clinical specimen.

Amplicon	Filtered reads			Primary cluster	
	Number of reads	Average length	Q-score	Number of reads	%^a^
RGN2	11,538	562.9	16.4	11,514	99.79
RGN3	5754	1464.3	16.0	5620	97.67
RGN4	4117	1945.2	16.0	4110	99.83
RGN5	3242	2625.0	15.9	3231	99.66

^a^Percentage of reads in the primary cluster with the highest read count.

**Table 6:** Identification of pathogenic fungi in human sputum using nanopore consensus sequencing.

Amplicon	Size (bases^a^)	Best BLAST hits		Number of			Identity (%)
		Taxon	Accession	Identical matches	Mis-matches	Gap opens	
RGN2	524	*Candida albicans*	NC_032096.1	522	1	1	99.62
RGN3	1429	*Candida albicans*	NC_032096.1	1428	0	1	99.93
RGN4	1914	*Candida albicans*	NC_032096.1	1911	1	2	99.84
RGN5	2591	*Candida albicans*	NC_032096.1	2588	1	2	99.88

^a^Length of the query consensus sequence.

## Discussion

Diagnostics of fungal infections have focused on a limited range of pathogens commonly encountered in clinical settings, where a single strain is thought to be responsible for the disease. Recent studies suggest the existence of mixed fungal colonization in the human body, and the resulting infection can lead to more severe symptoms than those caused by a single species^31^. With the continuing expansion of the number and diversity of fungal species potentially associated with human diseases, there has been an increasing demand for rapid and reliable means for analyzing the human fungal microbiota across broad taxonomic scales^32^.

The ITS region has been frequently used for the genetic identification of fungi in combination with high-throughput sequencing technologies^33^. Another candidate target for fungal identification includes the LSU rRNA gene^11^. However, both of these regions have taxonomic resolution limitations, and sequencing only the ITS or part of the LSU region can result in ambiguous taxonomic assignments^4,14^. Regarding sequencing technologies, although Sanger sequencing is still the standard method offering high sequence accuracy, it has several shortcomings for genetic species identification. It produces a single sequence with a maximum length of approximately 800 bases, resulting in low throughput and poor taxonomic resolution. Furthermore, the Sanger method cannot be directly applied to mixed complex samples. Due to its inability to resolve mixed amplicons derived from multiple species, it is required to obtain culture isolates or cloned DNA prior to sequencing. While high-throughput sequencing technologies could solve the issues concerning the analysis of mixed samples, the Illumina sequencing platform generates sequencing reads up to 600 bp in length, which fail to analyze longer genomic targets with the potential for higher discriminatory power. In the current study, we aimed to develop a practical sequencing approach utilizing a nanopore long-read sequencer to acquire contiguous sequence data encompassing both the ITS and LSU regions. We selected commonly used universal primers for fungal metabarcoding to amplify five target regions of varying length. Five primer sets (one shared forward primer with five reverse primers) amplified the target regions from 11 fungal species of different lineages, suggesting excellent taxonomic coverage of the primers used in this study. The LSU region downstream of the Rv5/RCA95m primer was not considered for sequencing because the nucleotide sequences were highly conserved among a broad range of fungal species (Supplementary Data S2).

Benchmarking with RGN1 that is often targeted for short-read sequencing analysis, we assessed the taxonomic resolution of long reads obtained by nanopore sequencing. To minimize the possibility of misidentification caused by sequencing errors, we aimed to provide a practical bioinformatics pipeline in which nanopore reads are clustered by similarity and are error-polished, yielding highly accurate consensus sequences. Pre-filtering the sequencing data at the minimum quality cutoff value of 15 removed poor-quality sequences that hampered effective read clustering^20,34^. With the similarity threshold optimized for each target region, nanopore sequencing data were successfully differentiated into the expected number of clusters (i.e., one cluster for the pure *Aspergillus niger* isolate and ten clusters for the mock community). The clustering conditions retained more than 96% of the reads for all the datasets, demonstrating the efficacy of our approach. A small number of contaminated reads that were erroneously demultiplexed were expected to be eliminated during this step. We observed that the optimum subread coverage for polishing was 300 reads. Considering the current per-read error rate for nanopore sequencing^28,35^, a smaller number of reads (e.g., 30) is insufficient for error correction, whereas an overly large read depth (e.g., 1000) could conversely decrease the consensus read accuracy. These results are supported by those of previous studies^34,36,37^, which showed that sequence accuracy can decrease when consensus sequences are built at coverage outside the intermediate subread number (approximately 300). Based on this, 300 randomly selected reads were used for read polishing. Consensus sequences were generated from each dataset, achieving a sequence accuracy ranging from 99.5% to 100%.

Using error-corrected consensus sequences, we assessed the discriminatory power of each target region to identify fungal species. We used the bit score difference between the top and second-best BLAST hits as an index to evaluate the accuracy and reliability of the taxonomic assignment. RGN1 provided the lowest taxonomic resolution for many of the fungal species assessed here, and the bit score differences were minimized with a few base differences between the top and second-best hits. As a result, taxonomic assignment using RGN1 is sensitive to a small number of errors that may arise from amplicon sequencing, leading to difficulties in distinguishing the target taxon from closely related species. These findings indicate that RGN1, which is widely utilized for fungal identification with short-read sequencing technologies, is more prone to misidentification. Nanopore sequencing technology enabled us to analyze longer amplicons (RGN3–5), including both ITS and LSU regions, providing a higher resolution for the taxonomic classification of fungi. The taxonomic resolution was almost comparable among RGN3, RGN4, and RGN5, indicating that part of the LSU, approximately the latter two-thirds of the region, was not sufficiently variable to improve the accuracy of the taxonomic classification of the target taxa. In general, increasing the amplification length negatively affects the PCR efficiency and amplicon yield. Furthermore, analyzing unnecessarily long amplicons is unfavorable because of the turnaround time required for both sequencing and data processing. Therefore, RGN3 could be an optimal target region for the rapid diagnosis of fungal infections, with an adequate sequence length to reliably discriminate a diverse range of fungal taxa^38^.

The clinical feasibility of the approach was confirmed using patient-derived samples. In accordance with our established procedure for DNA extraction from clinical specimens^20,39^, sputum samples were processed for cell lysis, followed by DNA purification using cellulose-based paramagnetic particles. Fungal rRNA genes were specifically amplified by PCR, targeting RGN2, RGN3, RGN4, and RGN5, albeit in the presence of a large amount of contaminating DNA of human origin. Nanopore amplicon sequencing and subsequent bioinformatic data processing identified *Candida albicans* as a candidate pathogen in each dataset. These results are consistent with those of the fungal culture test, demonstrating the reliability of our method for rapid pathogen detection. Thus, sequencing of the ITS–LSU region proved useful in the clinical context, allowing the identification of fungi with a higher taxonomic resolution.

Although our findings demonstrated the marked potential of long-read sequencing for the accurate taxonomic classification of fungal species, there were several limitations that affected this study. First, the current method employing consensus sequence generation from clustered nanopore reads is not suitable for handling samples with highly complex microbiota compositions. Our approach is effective in identifying particular microorganisms in samples containing less complex microbiota, such as diagnostic specimens for infectious diseases. Furthermore, it demonstrated high performance in identifying individual species within mixed communities with significant taxonomic variation, even at 85%–89% sequence identity thresholds. However, this method is not practical for segregating reads derived from closely related species because they cluster together at such low similarity thresholds^40^. Continuous improvements in nanopore sequencing chemistry will provide higher raw read and consensus sequence accuracies, allowing species-level resolution to characterize complex microbial communities. Another limitation is that several fungal species were not correctly classified by nanopore long-read sequencing targeting the ITS and LSU regions. For *Penicillium* and *Cryptococcus*, the entire ITS–LSU region did not provide species-level resolution. Depending on the fungal species, different universal genetic markers have been used for accurate taxonomic classification. The intergenic spacer (IGS) region within the rRNA operon has been proposed as a viable genetic marker for identifying individual species belonging to the genus *Cryptococcus*^41^. The entire fungal rRNA operon and other genetic loci may also serve as alternative targets for fungal metabarcoding^14,42^. Finally, the accuracy of taxonomic identification is highly dependent on the quality of the reference database. Shortcomings of the database include biases in taxonomic coverage, incomplete genome assembly, sequence errors in individual data, and inaccurate taxonomic annotations. These factors can cause erroneous taxonomic identifications^14,43^. To ensure precise and accurate identification of fungal species across broad phylogenetic lineages, concerted global efforts are required to create and maintain curated databases of the highest quality and scale.

## Conclusions

We present a streamlined workflow that utilizes nanopore long-read sequencing technology coupled with bioinformatics pipelines for the molecular identification of fungi from clinical specimens. Our approach provides a practical measure for the rapid diagnosis of infectious diseases with a high level of taxonomic resolution across a broad range of fungal species.

## Materials and methods

### Fungal genomic DNA

DNA extracted from *Aspergillus niger* (kindly provided by Dr. Takamitsu Imoto, Medical Research Institute, Kitano Hospital, Osaka, Japan) and a mock community DNA standard (Mycobiome Genomic DNA Mix, MSA-1010; ATCC, Manassas, VA, USA) were used to evaluate the validity of the sequencing methodology. The mock community standard was comprised of a mixture of DNA prepared from the following ten species: *Aspergillus fumigatus*, *Penicillium chrysogenum*, *Trichophyton interdigitale*, *Fusarium keratoplasticum*, *Candida albicans*, [*Candida*] *glabrata*, *Saccharomyces cerevisiae*, *Cryptococcus neoformans*, *Cutaneotrichosporon dermatis*, and *Malassezia globosa*. Due to the unclear rRNA gene copy number of each species, it was not possible to predict the relative abundance of individual fungal taxa based on sequencing read counts.

### Ethics approval and consent to participate

This study was approved by the Medical Ethics Committee of Osaka Red Cross Hospital (No. 894) and performed in accordance with the relevant guidelines and regulations. Written informed consent was obtained from all the participants.

### DNA extraction from a clinical specimen

DNA was extracted from a sputum sample obtained from a patient diagnosed with pneumonia, as previously described^20,39^. As a negative control, Dulbecco’s phosphate-buffered saline (Nacalai Tesque, Kyoto, Japan) was used as the solvent for sample collection and processed according to the same procedure in all subsequent experiments. The samples were subjected to mechanical cell disruption by bead beating using EZ-Beads (Promega, Madison, WI, USA), followed by DNA purification using a Maxwell RSC Blood DNA kit (Promega).

### Acquisition of fungal rRNA gene sequences

The genome sequence data for the fungal species of interest were obtained from the National Center for Biotechnology Information (NCBI) genome database (https://www.ncbi.nlm.nih.gov/data-hub/genome/; accessed on October 18, 2022), with taxonomic data and assembly accession numbers provided in Supplementary Table S1. To determine the amplicon length for fungal rRNA genomic regions, the primer sequences were searched against the genome assemblies using SeqKit software version 2.3.0 (https://bioinf.shenwei.me/seqkit/)^44^ with the command (italics within square brackets indicate inputs from a user that should be set appropriately): seqkit locate [*FASTA file*] -i -d -p [*primer sequence*]. The expected amplicon sizes were determined locating the positions of the primers used for each species (Supplementary Tables S2 and S3). The ITS–LSU region sequences were extracted and saved using the following command (Supplementary Data S1): seqkit subseq -r [*starting position*:*ending position*] [*FASTA file*] -w 0 > [*output file*]. Multiple sequence alignments were performed using MUSCLE version 3.8 (https://www.ebi.ac.uk/Tools/msa/muscle/)^45^ (Supplementary Data S2).

### PCR amplification of fungal rRNA genomic regions

The detailed protocol for nanopore amplicon sequencing, which is available elsewhere^20,46^, involved performing the first PCR with a single forward primer (Fw1) in combination with five different reverse primers (Rv1–5) to amplify the fungal rRNA gene regions (RGN1–5). The primers were designed based on those established in previous studies^23–27^, with slight modifications to avoid mismatches, and were synthesized with a 5′ tail containing the nanopore anchor sequences (Table 1). The reaction contained 5 ng (*Aspergillus niger* isolates and the mock community) or 20 ng (sputum sample) of template DNA, 12.5 µl of 2 × KAPA2G Robust HotStart ReadyMix (Kapa Biosystems, Wilmington, MA, USA), and 0.2 µM of each primer in a total volume of 25 µl. PCR was performed using a Veriti Thermal Cycler (Thermo Fisher Scientific, Waltham, MA, USA) with the following conditions: initial denaturation at 95 °C for 3 min, followed by 25 cycles of 95 °C for 15 s, 55 °C for 15 s, and 72 °C for 30 s.

### Sequencing library preparation

The amplified DNA was subjected to a second round of PCR using the PCR Barcoding Kit (Oxford Nanopore Technologies, Oxford, UK), which added a sample-specific barcode and a 5′ tag required for adapter attachment to the amplicons. The reaction contained 1 µl of first PCR products, 12.5 µl of 2 × KAPA2G Robust HotStart ReadyMix, and 0.5 µl of a barcoded primer pair in a total volume of 25 µl. Each primer pair contains a specific barcode of 24 bases for multiplexing up to 12 samples (https://community.nanoporetech.com/technical_documents/chemistry-technical-document/v/chtd_500_v1_revaj_07jul2016/barcoding-kits). PCR was conducted with the following conditions: initial denaturation at 95 °C for 3 min, followed by 8 cycles of 95 °C for 15 s, 62 °C for 15 s, and 72 °C for 30 s. The secondary PCR products were purified using AMPure XP beads (Beckman Coulter, Brea, CA, USA) at a ratio of 0.8 × (for RGN1 and RGN2) or 0.5 × (for RGN3–5). The samples were eluted with TN buffer (10 mM Tris–HCl, pH 8.0, 50 mM NaCl) and quantified using the QuantiFluor ONE dsDNA System (Promega). The resulting barcoded DNA samples were combined in approximately equimolar amounts (a total of 100 femtomoles in 10 µl of TN buffer). Rapid adapters (1 µl) were added and incubated at room temperature for 5 min. The sequencing sample for loading was prepared by mixing the pooled library (11 µl) with 34 μl of Sequencing Buffer, 25.5 μl of Loading Beads, and 4.5 μl of water to a final volume of 75 µl.

### Nanopore sequencing

The sequencing library was loaded onto an R9.4.1 Flow Cell (Oxford Nanopore Technologies) that was pre-primed with a Flow Cell Priming Kit (Oxford Nanopore Technologies). Sequencing runs were conducted on a MinION Mk1C device using MinKNOW software version 22.05.06. Raw data underwent base-calling and demultiplexing using the embedded toolkit Guppy version 6.1.5. Barcodes and adapter sequences were trimmed to generate pass reads saved in the FASTQ format. Data processing parameters included high-accuracy base-calling model, with trim barcodes set to “on”, require barcodes at both ends set to “off”, and detect mid-strand barcodes set to “on”. Typically, the pooled libraries were sequenced for three hours, generating approximately one billion bases of data per run.

### Sequence data processing

The exact pipeline commands are provided in the Supplementary Methods. The pipelines were run on an iMac Pro 2017 model (Apple, Cupertino, CA, USA) with 3.2 GHz 8-core Intel Xeon W-2140B processor, 64 GB RAM, and MacOS Monterey version 12.5.1. The FASTQ files were processed using the following steps. The italics within square brackets in the operation examples indicate the inputs from a user that should be set appropriately.

### Pre-filtering of reads

Reads were filtered by length to exclude those outside the expected size range defined for each target region: 220–530 bases for RGN1, 530–940 bases for RGN2, 1420–1880 bases for RGN3, 1890–2380 bases for RGN4, and 2550–3100 bases for RGN5, based on the predicted amplicon size distribution provided in Supplementary Table S3. A maximum indel error rate approximately 3% for nanopore sequencing was considered^28,35^. High-quality reads with a minimum quality score of 15, corresponding to an accuracy of approximately 97%, were selected. Read filtering was executed using SeqKit with the following command: seqkit seq -m [*minimum length*] -M [*maximum length*] -Q 15 [*FASTQ file*] > [*output file*]. Sequence reads containing simple repetitive sequences were eliminated by specifying the following search patterns (20 stretches of bases): aaaaaaaaaaaaaaaaaaaa, cccccccccccccccccccc, acacacacacacacacacac, agagagagagagagagagag, atatatatatatatatatat, and cgcgcgcgcgcgcgcgcgcg. The sequence patterns were saved as a text file (one record per line), and both the positive and negative strands were searched using SeqKit. The command is as follows: seqkit grep -vsif [*pattern file.txt*] [*FASTQ file*] > [*output file*]. Read statistics were obtained using the command seqkit stats [*FASTQ file*]. The average quality score was assessed as follows: seqkit fx2tab [*FASTQ file*] -q -n -i | awk '{sum +  = $2} END {print sum/NR}'.

### Clustering of reads

Similarity-based read clustering was performed using VSEARCH version 2.21.1 (https://github.com/torognes/vsearch)^47^. The reads were sorted by decreasing sequence length and clustered using the --id option with similarity thresholds of 85% for RGN1 and RGN2, 88% for RGN3 and RGN4, and 89% for RGN5. The command is: vsearch --cluster_fast [*FASTQ file*] --id [*pairwise identity*] --strand both --clusters [*output file*].

### Extraction of a centroid per cluster

A centroid sequence file (representative sequences per cluster) was created as follows. The reads in each cluster file were split into two parts using the command: seqkit split -p 2 [*FASTA file*]. This command generates two FASTA files named with part numbers (part_001 and part_002). The last read of the part_001 file was extracted with the following command: seqkit range -r -1:-1 [*cluster_part_001.fasta*] -w 0 > [*centroid.fasta*]..

### Random subsampling

To optimize the read depth for consensus calling, varying numbers of reads (30, 100, 300, 500, and 1000) were randomly selected ten times from the data sets of *Aspergillus niger*. Ten replicate read subsets were created by specifying different seed values (11, 22, 33, 44, 55, 66, 77, 88, 99, and 111) for random sampling using the following command: seqkit shuffle -s [*seed value*] [*cluster.fasta*] | seqkit head -n [*number*] -w 0 > [*subread.fasta*]. A sub-read coverage of 300 reads was used to analyze mock and clinical specimens.

### Consensus calling

Consensus sequences were generated using Medaka version 1.6.1 (https://github.com/nanoporetech/medaka). For sequence error correction, a centroid was used as a draft sequence for polishing with the randomly subsampled reads using the following command: medaka_consensus -d [*centroid.fasta*] -i [*subread.fasta*] -o [*output directory*] -m [*model*]. The resulting consensus sequences were trimmed of the primers and used for taxonomic assignment.

### Database

Reference and representative fungal genome assemblies were downloaded from the NCBI Genome (https://www.ncbi.nlm.nih.gov/data-hub/genome/). These included 453 RefSeq genomes (accessed October 25, 2022) and 3894 GenBank genomes (accessed October 26, 2022), which are listed in Supplementary Tables S5a and S5b, respectively. The RefSeq genome assembly of *Aspergillus niger* (GCF_000002855.3) was used as a database to evaluate the accuracy of the consensus sequences generated at a given subread coverage. The custom BLAST databases were built using the following command: makeblastdb -in [*assemblies.fna*] -out [*database title*] -dbtype nucl -hash_index -parse_seqids.

### Taxonomic classification

The consensus sequences were aligned against the fungal genome database using BLAST + version 2.13.0 (https://blast.ncbi.nlm.nih.gov/Blast.cgi). The BLAST search results were saved using the default pairwise (format 0) or custom tabular view options (format 6) (Supplementary Data S3–S6). The command for outputting results in format 0: blastn -query [*consensus.fasta*] -db [*database title*] -out [*output file*]. The command for outputting results in format 6: blastn -query [*consensus.fasta*] -db [*database title*] -out [*output file*] -outfmt "6 saccver qcovs bitscore nident mismatch gaps pident evalue". For each consensus sequence, the BLAST hits with the highest bit scores were assigned to the query. Spurious results assigning queries to phylogenetically distant species were excluded, and in cases where RefSeq and GenBank contents were available, the RefSeq genome assembly had priority. The bit score difference between the top two hits was calculated. Consensus calling errors were profiled by counting identical bases and errors (mismatches, insertions, and deletions) between query consensus sequence and the reference genome, based on the BLAST alignment results. The error rates at a given read depth were represented as the number of errors per 100 bases. The accuracy of the consensus sequences (BLAST identity) was defined as the number of sequence matches divided by the sum of matches, mismatches, insertions, and deletions in the alignment.

### Statistical analysis

Spearman's rank correlation coefficient was computed using GraphPad Prism version 9.5.1 (GraphPad Software, La Jolla, CA, USA) to compare the compositions of the mock communities analyzed in different sequencing regions. NMDS plots based on the Bray–Curtis dissimilarity indices was generated using R version 4.2.3 with the vegan package version 2.6–4 (https://github.com/vegandevs/vegan). Statistical significance was defined as a *P* value < 0.05.

## Supplementary Information

Supplementary Information.

## Data Availability

Filtered sequence data are available in the DDBJ DRA database (https://www.ddbj.nig.ac.jp/dra/index-e.html) under accession numbers DRR454434–DRR454447.
