# Supplementary material for: Using nanopore sequencing to identify fungi from clinical samples with high phylogenetic resolution
Source: Sci Rep. 2023 Jun 16;13:9785. doi: 10.1038/s41598-023-37016-0 (PMC10275880; doi:10.1038/s41598-023-37016-0)
Supplement: Supplementary file 1 — Supplementary Information. [file 41598_2023_37016_MOESM1_ESM.zip › Supplementary_Information/Supplementary_Data/Data_S2_align.pdf]

Supplementary Data S2. Multiple sequence alignment

```
Aspf:   Aspergillus fumigatus
Aspn:   Aspergillus niger
Cana:   Candida albicans
Cang:   [Candida] glabrata
Cryn:   Cryptococcus neoformans
Cutd:   Cutaneotrichosporon dermatis
Fusk:   Fusarium keratoplasticum
Malg:   Malassezia globosa
Penc:   Penicillium chrysogenum
Sacc:   Saccharomyces cerevisiae

                     ITS1 primer
Fusk            TAGAGGAAGTAAAAGTCGTAACAAGGTCTCCGTTGGTGAACCAGCGGAGGGATCATTACC
Penc            TAGAGGAAGTAAAAGTCGTAACAAGGTTTCCGTAGGTGAACCTGCGGAAGGATCATTACC
Aspf            TAGAGGAAGTAAAAGTCGTAACAAGGTTTCCGTAGGTGAACCTGCGGAAGGATCATTACC
Aspn            TAGAGGAAGTAAAAGTCGTAACAAGGTTTCCGTAGGTGAACCTGCGGAAGGATCATTACC
Cana            TAGAGGAAGTAAAAGTCGTAACAAGGTTTCCGTAGGTGAACCTGCGGAAGGATCATTACT
Cang            TAGAGGAACTAAAAGTCGTAACAAGGTTTCCGTAGGTGAACCTGCGGAAGGATCATTAAA
Sacc            TAGAGGAACTAAAAGTCGTAACAAGGTTTCCGTAGGTGAACCTGCGGAAGGATCATTAAA
Malg            TAGAGGAAGTAAAAGTCGTAACAAGGTTTCTGTAGGTGAACCTGCAGAAGGATCATTAGT
Cryn            TAGAGGAAGTAAAAGTCGTAACAAGGTTTCCGTAGGTGAACCTGCGGAAGGATCAGTAGA
Cutd            TAGAGGAAGTAAAAGTCGTAACAAGGTTTCCGTAGGTGAACCTGCGGAAGGATCATTAGT
                ******** ***************** ** ** ******** ** ** ****** **

Fusk            GAGTTA------------------------------------------------------
Penc            GA----------------------------------------------GTGAGGGCCCTC
Aspf            GA----------------------------------------------GTGAGGGCCCTC
Aspn            GA----------------------------------------------GTGCGGGTCCTT
Cana            GATTTGC-----------------------------------------------------
Cang            GAAATTTAATTGATTTGT-------------------CTGAGCTCGGAGAGAGACATCTC
Sacc            GAAATTTAATAATTTTGAAAATGGATTTTTTTGTTTTGGCAAGAGCATGAGAGCTTTTAC
Malg            GAAGATTCAAGGGCCAGC---------------CATACAGACGTACAATAAGTGTGTCTC
Cryn            GAATATT-----------------------------------------------GGACTT
Cutd            GAATTGC-------------------------------------------------TCTT
                **

Fusk            ------------------------------------------------------------
Penc            TGGG--------------------------------------------------------
Aspf            TGGG--------------------------------------------------------
Aspn            TGGG--------------------------------------------------------
Cana            ------------------------------------------------------------
Cang            TGGGGAGGACCAGTGTAGACACTCAGGAGGCTCCTAAAATATTTTCTCTGCTGTGAATGC
Sacc            TGGGCAAGAAGACAAGAGATGGAGAGT---------------------------------
Malg            TGGCGGCTCGTATCCACTA-----------------------------------------
Cryn            TGGTCCATTTA-------------------------------------------------
Cutd            TGAGCGTTAAAC------------------------------------------------


Fusk            ------------------------------------------------------------
Penc            ------------------------------------------------------------
Aspf            ------------------------------------------------------------
Aspn            ------------------------------------------------------------
Cana            ------------------------------------------------------------
Cang            TATTTCTCCTGCCTGCGCTTAAGTGCGCGGTTGGTGGGTGTTCTGCAGTGGGGGGAGGGA
Sacc            --CCAGCCGGGCCTGCGCTTAAGTGCGCGGTCTTGCTAGGCTTGTAAGTTTCTTTCTTGC
Malg            ------------------------------------------------------------
Cryn            ------------------------------------------------------------
Cutd            ------------------------------------------------------------


Fusk            ---------------------------------------------------------TAC
Penc            ---------------------------------------------------------TCC
Aspf            ---------------------------------------------------------TCC
Aspn            ---------------------------------------------------------CCC
Cana            ---------------------------------------------------------TTA
Cang            GCCGACAAAGACCTGGGAGTGTGCGTGGATCTCTCTATTCCAAAGGAGGTGTTTTATCAC
Sacc            TATTCCAAACGGTGAGAGATTTCTGTGCTTTTGTT---------------------ATAG
Malg            ---------------------------------------------------------TAC
Cryn            ---------------------------------------------------------TCT
Cutd            ---------------------------------------------------------TAT


Fusk            AACTCATCAACCCTGTGAACATACCTAAAACG----------------------------
Penc            AACCTCCCACCCGTGTTTAT---TTTACCTTG----------------------------
Aspf            AACCTCCCACCCGTGTCTAT---CGTACCTTG----------------------------
Aspn            AACCTCCCATCCGTGTCTAT---TGTACCCTG----------------------------
Cana            ATTGCACCACATGTGTTTTTCTTTGAAACAAAC---------------------------
Cang            ACGACTCGACACTTTCTAAT---TACTACACACAGTGGAGTTTACTTTACTACTATTCTT
Sacc            GACAATTAAAACCGTTTCAA---TACAACACACTGTGGAGTTTTCATATCTTTGCAACTT
Malg            ATCCATAAACCCGTGTGCAC---TGTTAAGGAGTAAGAAAGAAGGGGAGGGAGAGAGTGC
Cryn            ACCCATCTACACCTGTGAAC---TGTTTAT------------------------------
Cutd            ATCCATCTACACCTGTGAAC---TGTTGAT------------------------------
                        *

Fusk            --TTGCTTCGGCGG-----------------------GAACAGACGGCCCCGTAACACGG
Penc            --TTGCTTCGGCGGGCCCGCCTTAACTGGCCGCCGGGGGGC------TTACGCCCCCGGG
Aspf            --TTGCTTCGGCGGGCCCGCCGTT--TCGACGGCCGCCGGGGAGGCCTTGCGCCCCCGGG
Aspn            --TTGCTTCGGCGGGCCCGCCGCTTGTCGGCCGCCGGGGGGGCGCCTCTGC-CCCCCGGG
Cana            --TTGCTTTGGCGG------------------------------------------TGGG
Cang            TTGTTCGTTGGGGG-----------------------------AACGCTCTCTTTCGGGG
Sacc            --TTTCTTTGGG----------------------------------------CATTCGAG
Malg            ATGTGCTTTG----------------------------CATATAACTCTCTCTCTTTCTC
Cryn            --GTGCTTCGGCACGTTTTA----------------------------------------
Cutd            --TGACTTCGGTCAATTACTTT--------------------------------------
                     * * *

Fusk            GCCG--CCCCCGCCAGAGGACC-----CCCTAACTCTGTTTCTATTATGTTTCTTCTGAG
Penc            CCCG--CGCCCGCCGAAGACACC----CTCGAACTCTGTCTGAA--GATTGTAGTCTGAG
Aspf            CCCG--CGCCCGCCGAAGACCCCAA--CATGAACGCTGTTCTGAAAGTATGCAGTCTGAG
Aspn            CCCG--TGCCCGCCGGAGACCCCAA--CACGAACACTGTCTGAAA-GCGTGCAGTCTGAG
Cana            CCCAGCCTGCCGCCAGAGGTCTAAACTTACAACCAATTTTTTATCAACTTGTCACACCAG
Cang            GGGA--GTTCTCCCAGTGGATGCAAA-CACAAACAAATATTTTTTTAAACTAATTC--AG
Sacc            CAAT--CGGGGCCCAGAGGTAACAAA-CACAAACAATTTTATCTATTCATTAAATTTTTG
Malg            TTCC--TTTCTCTCTCTGGTTAATTA-CACAAACTCGTATGGATTTGTATG-AACGTGAG
Cryn            ---------------------------CACAAACT--------TCTAAATGTAATGAATG
Cutd            ---------------------------TACAAACATT-----------GTGTAATGAACG
                               * *                         *

Fusk            TAAA---------------ACAAGC-------------AAATAAAT-----------TAA
Penc            TGAA----------------AATAT-------------AAATTATT-----------TAA
Aspf            TTGA---------------TTATCG-------------TAATCAGT-----------TAA
Aspn            TTGA--------------TTGAATG-------------CAATCAGT-----------TAA
Cana            ------------------ATTATTA-------------CTAATAGT-----------CAA
Cang            TCAA-CACAAGATTTCTTTTAGTAG-------------AAAACAACTT---------CAA
Sacc            TCAAAAACAAGAAT---TTTCGTAA-------------CTGGAAATTTTAAAATATTAAA
Malg            ATAT--------------ATCGTTGGACCGTCACTGGCCAACAAATATAA-------CAC
Cryn            TAAT-----------CATATTATAA-------------CAATAA-------------TAA
Cutd            TCAT--------------GTTATTA-------------TAACAA-------------AAT
                                                           *              *
                                      ITS2 primer
Fusk            AACTTTCAACAACGGATCTCTTGGCTCTGGCATCGATGAAGAACGCAGCGAAATGCGATA
Penc            AACTTTCAACAACGGATCTCTTGGTTCCGGCATCGATGAAGAACGCAGCGAAATGCGATA
Aspf            AACTTTCAACAACGGATCTCTTGGTTCCGGCATCGATGAAGAACGCAGCGAAATGCGATA
Aspn            AACTTTCAACAATGGATCTCTTGGTTCCGGCATCGATGAAGAACGCAGCGAAATGCGATA
```

```
Cana            AACTTTCAACAACGGATCTCTTGGTTCTCGCATCGATGAAGAACGCAGCGAAATGCGATA
Cang            AACTTTCAACAATGGATCTCTTGGTTCTCGCATCGATGAAGAACGCAGCGAAATGCGATA
Sacc            AACTTTCAACAACGGATCTCTTGGTTCTCGCATCGATGAAGAACGCAGCGAAATGCGATA
Malg            AACTTTCGACAACGGATCTCTTGGTTCTCCCATCGATGAAGAACGCAGCGAAACGCGATA
Cryn            AACTTTCAACAACGGATCTCTTGGCTTCCACATCGATGAAGAACGCAGCGAAATGCGATA
Cutd            AACTTTCAACAACGGATCTCTTGGCTCTCGCATCGATGAAGAACGCAGCGAAATGCGATA
                ******* **** *********** *    ********************** ******

Fusk            AGTAATGTGAATTGCAGAATTCAGTGAATCATCGAATCTTTGAACGCACATTGCGCCCGC
Penc            CGTAATGTGAATTGCA-AATTCAGTGAATCATCGAGTCTTTGAACGCACATTGCGCCCCC
Aspf            AGTAATGTGAATTGCAGAATTCAGTGAATCATCGAGTCTTTGAACGCACATTGCGCCCCC
Aspn            ACTAATGTGAATTGCAGAATTCAGTGAATCATCGAGTCTTTGAACGCACATTGCGCCCCC
Cana            CGTAATATGAATTGCAGATATTCGTGAATCATCGAATCTTTGAACGCACATTGCGCCCTC
Cang            CGTAATGTGAATTGCAGAATTCCGTGAATCATCGAATCTTTGAACGCACATTGCGCCCTC
Sacc            CGTAATGTGAATTGCAGAATTCCGTGAATCATCGAATCTTTGAACGCACATTGCGCCCCT
Malg            GGTAATGTGAATTGCAGAATTCCGTGAATCATCGAATCTTTGAACGCACCTTGCGCTCTA
Cryn            AGTAATGTGAATTGCAGAATTCAGTGAATCATCGAGTCTTTGAACGCAACTTGCGCCCTT
Cutd            AGTAATGTGAATTGCAGAATTCAGTGAATCATCGAATCTTTGAACGCAACTTGCGCTCTC
                  **** ********* *  *  ************ ************  ****** *

Fusk            CAGTATTCTGGCGGGCATGCCTGTTCGAGCGTCATTACAACCCTCAGGCCCCCGGGCCT-
Penc            TGGTATTCCGGGGGGCATGCCTGTCCGAGCGTCATTTCTGCCCTCAAGCAC---GGCTTG
Aspf            TGGTATTCCGGGGGGCATGCCTGTCCGAGCGTCATTGCTGCCCTCAAGCAC---GGCTTG
Aspn            TGGTATTCCGGGGGGCATGCCTGTCCGAGCGTCATTGCTGCCCTCAAGCCC---GGCTTG
Cana            TGGTATTCCGGAGGGCATGCCTGTTTGAGCGTCGTTTCT-CCCTCAAACCGCTGGGTTT-
Cang            TGGTATTCCGGGGGGCATGCCTGTTTGAGCGTCATTTCC-TTCTCAAACACATTGTGTTT
Sacc            TGGTATTCCAGGGGGCATGCCTGTTTGAGCGTCATTTCC-TTCTCAAACATTCTG--TTT
Malg            TGGTATTCCGTAGAGCATGCCTGTTTGAGTGCCGTGAATTCTCCCATCCCAAGCGGTTT-
Cryn            TGGTATTCCGAAGGGCATGCCTGTTTGAGAGTCATGAAA-ATCTCAATCCCTCGGGTTTT
Cutd            TGGTATTCCGGAGAGCATGCCTGTTTGAGTATCATGAAA--TCTCAACCATTAGGGTTTC
                  ******    * **********  ***   * *       * **  *     *   *

Fusk            GGCGTTGGGGATCGGCGAGGCGCCCCCTGCGGGCACACGCCGTCCCCCAAATACAGTGGC
Penc            TGTGTTGGGCCC--------CGTCCTCCGATCCCGGGGGACGGGCCCGAAAGGCAGCGGC
Aspf            TGTGTTGGGCCCC-------CGTCCCCCTCTCCCGGGGGACGGGCCCGAAAGGCAGCGGC
Aspn            TGTGTTGGGTCGC-------CGTCCCCCTCTCCGGGGGGACGGGCCCGAAAGGCAGCGGC
Cana            GGTGTTGAGCAATA------CGACTTGGGTTT-----------GCTTGAAAGACGGTAGT
Cang            GGTAGTGAG-----------TGATACTCGTTTTTGAGT--TAACTTGAAATTGTAGGCCA
Sacc            GGTAGTGAG-----------TGATACT--CTTTGGAGT--TAACTTGAAATTGCTGGCCT
Malg            -TTATCAAAGAATTGCTAGGCGA----------------AGGGGTTGAGATGGGCGTTGT
Cryn            ATTACCTGT-------------------------------TGGACTTGGATTTGGGTGTT
Cutd            TTAAT-------------------------------------GGCTTGGATTTGGGCGCT
                                                         *     *

Fusk            GGT------------------------------------CCCGC-----------CGCAG
Penc            GGC------------------------------------ACCGC-----------GTCCG
Aspf            GGC------------------------------------ACCGC-----------GTCCG
Aspn            GGC------------------------------------ACCGC-----------GTCCG
Cana            GGT------------------------------------AAGGC---------GGGATCG
Cang            TATCAGTATGTGGGACACGAGCGCAAGCTTCTCTATTAATCTGC----------TGCTCG
Sacc            TTTCATTGGATGTTTTTTTTCCAAAGAGAGGTTTCTCTGCGTGC------TTGAGGTATA
Malg            TATAACTGCTTTTCTTCTCTAGAAAAGCTCGCCCGAAAAACAGCTAACGCCTCTGGGCCA
Cryn            TGC-------------------------------CGCGACCTGCAAAGGACGTCGGCTCG
Cutd            GCC-----------------------------------ACTTGC--------CTGGCTCG
                                                          **

Fusk            CTTCCATTGCGTA-----------------------GTAGCTAACACCT-----------
Penc            GTCCTCGAGCGTA--------------------TGGGGCTTTGTCACCC-----------
Aspf            GTCCTCGAGCGTA--------------------TGGGGCTTTGTCACCT-----------
Aspn            ATCCTCGAGCGTA--------------------TGGGGCTTTGTCACAT-----------
Cana            CTTTGACAATGGC--------------------TTAGGTCTAACCAAAA-----------
Cang            TTT---GCGCGAGCGGCGGGGGTTAATACTGTATTAGGTTTTACCAACT-----------
Sacc            ATGCAAGTACGGT----------------CGTTTTAGGTTTTACCAACT-----------
Malg            CTTTGCATCCGCTTCTCTGAGGGGAGAAGCGGCCAAGCGCGCTCTGATCATCAGGCATAG
Cryn            CCTTAAATGTGTT------------------AGTGGGAAGGTGATTACCTGTCAGCCCGG
Cutd            CCTTAAAAGAGTT------------------------AGCGTATTAACTTGTCGATCTGG
                          *

Fusk            ------------------------------------CGCAACTGGAGAGCG---------
Penc            -----------------------------------GCTCTGTAGGCCCG-----------
Aspf            -----------------------------------GCTCTGTAGGCCCGGC---------
Aspn            -----------------------------------GCTCTGTAGGATTGGC---------
Cana            -----------------------------------ACATTGCTTGCG-------------
Cang            ------------------------------------CGGTGTTGATCTAGGGAGGGATAA
Sacc            -GCGGCTAATCTTTTTTTATACTGAGCGTATTGGAACGTTATCGATAAGAAGA-------
Malg            CATGATACGTCATTTGCT-----------------ATGCTGTAGGAGAGCATTTGGTTGT
Cryn            CGTAATAAGTTTCGCTG------------------GGCCTATGGGGTAG-----------
Cutd            CGTAATAAGTTTCG---------------------CTGGTGTAGACTTGAGAA-------


Fusk            -----------GCGCGGCCATGCCGTAAAAACACCC----A----------ACTTCTGAA
Penc            ---------------GCCGGCGCTTGCCGATCAACCCAAAT----------TTTTATCCA
Aspf            --------------CGGCGCCAGCCGACACCCAACT--TTA----------TTTTTCTAA
Aspn            -----------CGGCGCCTGCCGACGTTTTCCAACC----A----------TTCTTTCCA
Cana            -------------GCGGTAACGTCCACCACGTAT-----------------ATCTTCAAA
Cang            GTGAGTGTTTTGTGCG--------TGCTGGGCAGAC--AGA----------CGTCTTTAA
Sacc            -----------GAGCG---------TCTAGGCGAAC--AAT----------GTTCTTAAA
Malg            GGTTATACCGCGTGCGTCATTTTTTTTTTTGCAAACGCAAAGAAAAAAGCCCCCTTTCAT
Cryn            -------------TCTTCGGCTTGCTGATAACAACC----A----------TCTCTTTTT
Cutd            -----------GTGCGCTTCTAATCGTCTTCGGACA---------------ATTCTTGAA

                                                     ITS4 primer
Fusk            TGTTGACCTCGAATCAGGTAGGAATACCCGCTGAACTTAAGCATATCAATAAGCGGAGGA
Penc            GGTTGACCTCGGATCAGGTAGGGATACCCGCTGAACTTAAGCATATCAATAAGCGGAGGA
Aspf            GGTTGACCTCGGATCAGGTAGGGATACCCGCTGAACTTAAGCATATCAATAAGCGGAGGA
Aspn            GGTTGACCTCGGATCAGGTAGGGATACCCGCTGAACTTAAGCATATCAATAAGCGGAGGA
Cana            CTTTGACCTCAAATCAGGTAGGACTACCCGCTGAACTTAAGCATATCAATAAGCGGAGGA
Cang            GTTTGACCTCAAATCAGGTAGGGTTACCCGCTGAACTTAAGCATATCAATAAGCGGAGGA
Sacc            GTTTGACCTCAAATCAGGTAGGAGTACCCGCTGAACTTAAGCATATCAATAAGCGGAGGA
Malg            TTCTGGTCTCAAATCAGGTAGGATCACCCGCTGAACTTAAGCATATCAATAAGCGGAGGA
Cryn            GTTTGACCTCAAATCAGGTAGGGCTACCCGCTGAACTTAAGCATATCAATAAGCGGAGGA
Cutd            CTCTGGTCTCAAATCAGGTAGGACTACCCGCTGAACTTAAGCATATCAATAAGCGGAGGA
                   **  ***  **********   ***********************************

Fusk            AAAGAAACCAACAGGGATTGCCCCAGTAACGGCGAGTGAAGCGGCAACAGCTCAAATTTG
Penc            AAAGAAACCAACAGGGATTGCCCCAGTAACGGCGAGTGAAGCGGCAAGAGCTCAAATTTG
Aspf            AAAGAAACCAACAGGGATTGCCTCAGTAACGGCGAGTGAAGCGGCAAGAGCTCAAATTTG
Aspn            AAAGAAACCAACCGGGATTGCCTCAGTAACGGCGAGTGAAGCGGCAAGAGCTCAAATTTG
Cana            AAAGAAACCAACAGGGATTGCCTCAGTAGCGGCGAGTGAAGCGGCAAAAGCTCAAATTTG
Cang            AAAGAAACCAACTGGGATTGCCTTAGTAACGGCGAGTGAAGCGGCAAAAGCTCAAATTTG
Sacc            AAAGAAACCAACCGGGATTGCCTTAGTAACGGCGAGTGAAGCGGCAAAAGCTCAAATTTG
Malg            AAAGAAACTAACAAGGATTCCCCTAGTAACGGCGAGCGAAGCGGGAAGAGCTCAAATTTG
Cryn            AAAGAAACTAACAAGGATTCCCTTAGTAACGGCGAGTGAACCGGGAAGAGCTCAAATTTG
Cutd            AAAGAAACTAACTAGGATTCCCTTAGTAACGGCGAGTGAACCGGGAAAAGCTCAAATTTG
                ******** ***  ***** **  **** ******* *** *** ** ************

Fusk            AAATCTGGC---TCTCGG--GCCCGAGTTGTAATTTGTAGAGGATGCTTTTGGTGAGGTG
Penc            AAAGCTGGC-TCCTTCGGG-GTCCGCATTGTAATTTGTAGAGGATGCTTCGGGAGCGGTC
Aspf            AAAGCTGGC-CCCTTCGGG-GTCCGCGTTGTAATTTGCAGAGGATGCTTCGGGTGCAGCC
Aspn            AAAGCTGGC-TCCTTCGGA-GTCCGCATTGTAATTTGCAGAGGATGCTTTGGGTGCGGCC
Cana            AAATCTGGC-GTCTTTGGC-GTCCGAGTTGTAATTTGAAGAAGGTATCTTTGGGCCCGGC
Cang            AAATCTGGT-ACCTTTGGT-GCCCGAGTTGTAATTTGGAGAGTACCACTTTGGGACTGTA
Sacc            AAATCTGGT-ACCTTCGGT-GCCCGAGTTGTAATTTGGAGAGGGCAACTTTGGGGCCGTT
Malg            AAAGCTGGC-ACCTCCGGT-GTCCGCGTTGTAATCTCGAGACG-TGTTTTCCGTGCGGCT
Cryn            AAATCTGGCGTCCTCCGGGCGTCCGAGTTGTAATCTACAGAAA-CGTTTTCCGTGCTGGA
Cutd            TAATCTGGCTGTCTTCGATAGTCCGAGTTGTAATCTATAGACG-TGTTTTCCGTGCTGGA
```

```
             ** ****        *   * ***  ******* *  ***       *   *    *

Fusk        CCTT--CCGAGTTCCCTGGAACGGGACGCCATAGAGGGTGAGAGCCCCGTC--TGGTT--
Penc        CC-CATCTAAGTGCCCTGGAACGGGACGTCATAGAGGGTGAGAATCCCGTA--TGGGATG
Aspf        CC-CGTCTAAGTGCCCTGGAACGGGCCGTCATAGAGGGTGAGAATCCCGTC--TGGGACG
Aspn        CC-CGTCTAAGTGCCCTGGAACGGGCCGTCAGAGAGGGTGAGAATCCCGTC--TTGGGCG
Cana        TCTTGTCTATGTTCCTTGGAACAGGACGTCACAGAGGGTGAGAATCCCGTG--CGATGA-
Cang        CTTTGCCTATGTTCCTTGGAACAGGACGTCATGGAGGGTGAGAATCCCGTG--TGGCGA-
Sacc        CCTTGTCTATGTTCCTTGGAACAGGACGTCATAGAGGGTGAGAATCCCGTG--TGGCGAG
Malg        CTATGGACAAGTCCCTTGGAATAGGGCATCGTAGAGGGTGAGAATCCCGTACTTGCCATG
Cryn        CCGTGTCTAAGTCCCTTGGAATAGGGTATCAAAGAGGGTGACAATCCCGTACTTGACAC-
Cutd        CCGTATCTAAGTCCCTTGGAACAGGGTATCAAAGAGGGTGACAATCCCGTGCTTGATAC-
                      ** ** *****  **    *   ******** *  *****

Fusk        GGACACCGATCCTC-TGTAAAGCTCCTTCGACGAGTCGAGTAGTTTGGGAATGCTGCTCT
Penc        GGGTGTCCGCGCCCGTGTGAAGCTCCTTCGACGAGTCGAGTTGTTTGGGAATGCAGCTCT
Aspf        GGGTGTCTGCGTCCGTGTGAAGCTCCTTCGACGAGTCGAGTTGTTTGGGAATGCAGCTCT
Aspn        GGGTGTCCGTGCCCGTGTAAAGCTCCTTCGACGAGTCGAGTTGTTTGGGAATGCAGCTCT
Cana        GATGACCCGGGTCTGTGTAAAGTTCCTTCGACGAGTCGAGTTGTTTGGGAATGCAGCTCT
Cang        GGGTGTCAGTTCTT-TGTAAAGGGTGCTCGAAGAGTCGAGTTGTTTGGGAATGCAGCTCT
Sacc        GAGTG-CGGTTCTT-TGTAAAGTGCCTTCGAAGAGTCGAGTTGTTTGGGAATGCAGCTCT
Malg        GAAGAACCGTGCTT-TGCGATACACGCTCTAAGAGTCGAGTTGTTTGGGATTGCAGCTCA
Cryn        GATCACCAGTGCTC-TGTGATACGTTTTCTACGAGTCGCGTTACTTGGGAGTGTAGCGCA
Cutd        GACCACCAGTGCTC-TGTGATACACGCTCTACGAGTCGAGTTGTTTGGGAATGCAGCTCA
            *     *        **  *       ** * ****** **   ****** **  ** *

Fusk        AAATGGGAGGTATATGTCTTCTAAAGCTAAATACCGGCCAGAGACCGATAGCGCACAAGT
Penc        AAATGGGTGGTAAATTTCATCTAAAGCTAAATATTGGCCGGAGACCGATAGCGCACAAGT
Aspf        AAATGGGTGGTAAATTTCATCTAAAGCTAAATACTGGCCGGAGACCGATAGCGCACAAGT
Aspn        AAATGGGTGGTAAATTTCATCTAAAGCTAAATACTGGCCGGAGACCGATAGCGCACAAGT
Cana        AAGTGGGTGGTAAATTCCATCTAAAGCTAAATATTGGCGAGAGACCGATAGCGAACAAGT
Cang        AAGTGGGTGGTAAATTCCATCTAAAGCTAAATACAGGCGAGAGACCGATAGCGAACAAGT
Sacc        AAGTGGGTGGTAAATTCCATCTAAAGCTAAATATTGGCGAGAGACCGATAGCGAACAAGT
Malg        AAATGGGTGGTAGACTCCATCTAAAGCTAAATATCGGGGAGAGACCGATAGCGAACAAGT
Cryn        AAATGGGTGGTAAACTCCATCTAAAGCTAAATATTGGTGGAAGACCGATAGCGAACAAGT
Cutd        AAATGGGTGGTAAATTCCATCTAAAGCTAAATATTGGCGAGAGACCGATAGCGAACAAGT
            ** **** **** *   * **************  **    ************ ******

Fusk        AGAGTGATCGAAAGATGAAAAGAACTTTGAAAAGAGAGTTAAACAGTACGTGAAATTGTT
Penc        AGAGTGATCGAAAGATGAAAAGCACTTTGAAAAGAGAGTTAAAAAGCACGTGAAATTGTT
Aspf        AGAGTGATCGAAAGATGAAAAGCACTTTGAAAAGAGAGTTAAACAGCACGTGAAATTGTT
Aspn        AGAGTGATCGAAAGATGAAAAGCACTTTGAAAAGAGAGTTAAACAGCACGTGAAATTGTT
Cana        ACAGTGATGGAAAGATGAAAAGAACTTTGAAAAGAGAGTGAAAAAGTACGTGAAATTGTT
Cang        ACAGTGATGGAAAGATGAAAAGAACTTTGAAAAGAGAGTGAAAAAGTACGTGAAATTGTT
Sacc        ACAGTGATGGAAAGATGAAAAGAACTTTGAAAAGAGAGTGAAAAAGTACGTGAAATTGTT
Malg        ACCGTGAGGGAAAGATGAAAAGCACTTTGGAAAGAGAGTTAAA-AGTACGTGAAATTGTC
Cryn        ACCGTGAGGGAAAGATGAAAAGCACTTTGGAAAGAGAGTTAAACAGTACGTGAAATTGTT
Cutd        ACCGTGAGGGAAAGATGAAAAGCACTTTGGAAAGAGAGTTAAACAGTACGTGAAATTGTT
            *  ****  ************* ****** ********* *** ** ************

Fusk        GAAAGGGAAGCGCTTGTGACCAGACTTGGGCTTGGTTGATCATCCGGGGTTCTCCCCG--
Penc        GAAAGGGAAGCGCTTGCGACCAGACTCGCTCGCGG-GGTTCAGCCGGCATTCGTGCCG--
Aspf        GAAAGGGAAGCGTTTGCGACCAGACTCGCCCGCGG-GGTTCAGCCGGCATTCGTGCCG--
Aspn        GAAAGGGAAGCGCTTGCGACCAGACTCGCCCGCGG-GGTTCAGCCGGCATTCGTGCCG--
Cana        GAAAGGGAAGGGCTTGAGATCAGACTTGGTATTTTGCATGCTGCTCTCTCGGGGGC----
Cang        GAAAGGGAAGGGCATTTGATCAGACATGGTGTTTTGCGCCCCTTGCCTCTCGTGGGCTTG
Sacc        GAAAGGGAAGGGCATTTGATCAGACATGGTGTTTTGTGCCCTCTGCTCCTTGTGGGTAGG
Malg        GAAAGGGAAGCACTTGAAGTCAGCCATGCTGCTTGAGACTCAGCCTGGCCTTTGGGCTTG
Cryn        GAAAGGGAAACGATTGAAGTCAGTCGTGTCTATTG-GGTTCAGCCAGTTCTGCTG-----
Cutd        GAAAGGGAAACGATTGAAGTCAGTCGTGTTCTTTG-GATTCAGCTAGTTCTTCTA-----
            *********     *     *** *  *            *

Fusk        -GTGCACTCTTCC-GGCCCAGGCCAGCATCAGTTCGCCCTGGGGGGACAAAGGCTTCGGG
Penc        -GTGTACTTCCCCGCGGGCGGGCCAGCGTCGGTTTGGGC-GGTCGGTCAAAGGCCCTCGG
Aspf        -GTGTACTTCCCCGTGGGCGGGCCAGCGTCGGTTTGGGC-GGCCGGTCAAAGGCCCTCGG
Aspn        -GTGTACTTCCCCGTGGGCGGGCCAGCGTCGGTTTGGGC-GGCCGGTCAAAGGCCCCTGG
Cana        -GGCCGCTGCGGT-TTACCGGGCCAGCATCGGTTTGGAG-CGGCAGGATAATGGCGGAGG
Cang        GGACTCTCGCAGC-TCACTGGGCCAGCATCGGTTTTGGC--GGCCGGAAAAAAACCTAGGG
Sacc        GGAATCTCGCATT-TCACTGGGCCAGCATCAGTTTTGGT--GGCAGGATAAATCCATAGG
Malg        -GTGTATTTCTCG-GTGGCAAGCCAGCATTGGTTTGGGT-CGTCGGAGAAGCGCATAGGG
Cryn        -GTGTATTCCCTT-TAGACGGGTCAACATCAGTTCTGAT-CGGTGGATAAGGGCTGGAGG
Cutd        -GTCTACTTCCAT-TGAACGGGTCAACATCAGTTTTGTC-CGGTGGATAAAGATAGTAGG
             *                   * ** * *  ***       *   *   *        **

Fusk        AATGTGG-----CTCTCCGG---GGAGTGTTATAGCCCGTTGCGTAATAC-CCTGTGGCG
Penc        AAGGTAACG---CCCCTAGG---GGCGTCTTATAGCCGAGGGTGCAATGCGACCTGCCTA
Aspf        AATGTATCA---CCTCTCGG---GGTGTCTTATAGCCGAGGGTGCAATGCGGCCTGCCTG
Aspn        AATGTAGTG---CCCTCCGG---GGCACCTTATAGCCAGGGGTGCAATGCGGCCAGCCTG
Cana        AATGTGGCACGGCTTCTGC----TGTGTGTTATAGCCTCTGA--CGATACTGCCAGCCTA
Cang        AATGTGGCTCTGCGCCTCGGTGTAGAGTGTTATAGCCCTGGG--GAATACGGCCAGTCGG
Sacc        AATGTAGCT---TGCCTCGG---TAAGTATTATAGCCTGTGG--GAATACTGCCAGCTGG
Malg        AATGTAG-----CGTCTCCG---GACGTGTTATAGCCTTATGCAGGATAC-GACGACCTG
Cryn        AATGTGGCA---CTCTTCGG---GGTGTGTTATAGCCTCCTGTCGCATAC-ACTGGTTGG
Cutd        AATGTAG-----CTCCCCCG---GGAGTGTTATAGACTATTATTGCATAC-ACTGGGTGA
            ** **                        ****** *         ** *

Fusk        GACTGAGGTTCGCGCATT-------------------------------CGCAAGGATGC
Penc        GACCGAGGAACGCGCTTC-------------------------------GGCTCGGACGC
Aspf        GACCGAGGAACGCGCTTC-------------------------------GGCTCGGACGC
Aspn        GACCGAGGAACGCGCTTC-------------------------------GGCACGGACGC
Cana        GACCGAGGACTGCGGTTTTT-----------------------------ACCTAGGATGT
Cang        GACCGAGGACTGCGATACTTGTT--------------------------ATCTAGGATGC
Sacc        GACTGAGGACTGCGACGT-----------------------------AAGTCAAGGATGC
Malg        GATCAAGGAACGCAGTGTGCCCTCTGGGCGGGTCTTCGGACACCTTCACACTTAGGATGC
Cryn        GACTGAGGAATGCAGCTCGCCTTTATGGCCGGGGTTCGCCCACGTTCGAGCTTAGGATGT
Cutd        GACTGAGGACTGCAGCTCGCCTT-TTGGCCGGTCTTCGGACACGTTCGAGCTTAGGATGT
            **   ***   **                                         *** *

Fusk        TGGCGTAATGGTCATCAGTGACCCGTCTTGAAACACGGACCAAGGAGTCGTCTTCGTATG
Penc        TGGCATAATGGTCGTAAACGACCCGTCTTGAAACACGGACCAAGGAGTCTAACATCTACG
Aspf        TGGCGTAATGGTCGTAAATGACCCGTCTTGAAACACGGACCAAGGAGTCTAACATCTACG
Aspn        TGGCATAATGGTCGTAAACGACCCGTCTTGAAACACGGACCAAGGAGTCTAACATCTACG
Cana        TGGCATAATGATCTTAAGTCGCCCGTCTTGAAACACGGACCAAGGAGTCTAACGTCTATG
Cang        TGGCATAATGGTTATATGCCGCCCGTCTTGAAACACGGACCAAGGAGTCTAACGTCTATG
Sacc        TGGCATAATGGTTATATGCCGCCCGTCTTGAAACACGGACCAAGGAGTCTAACGTCTATG
Malg        TGGCGTAATGGCTTTAAGTGGCCCGTCTTGAAACACGGACCAAGGAGTCTAACATGTGTG
Cryn        TGACAAAATGGCTTTAAACGACCCGTCTTGAAACACGGACCAAGGAGTCTAACATATCTG
Cutd        TGACATAATGGCTTTAAACGACCCGTCTTGAAACACGGACCAAGGAGTCTAACATATCTG
            ** *  ****    *      ****************************       *  *

Fusk        CGAGTGTTCGGGTGTCAAACCCCTACGCGAAATGAAAGTGAACGCAGGTGAGAGCTTC--
Penc        CGAGTGTTCGGGTGTCAAACCCGTGCGCGAAGTGAAAGCGAACGGAGGTGGGAACCCT--
Aspf        CGAGTGTTCGGGTGTCAAACCCGTACGCGCAGTGAAAGCGAACGGAGGTGGGAGCCCC--
Aspn        CGAGTGTTCGGGTGTCAAACCCGTGCGCGCAGTGAAAGCGAACGGAGGTGGGAGCCCC--
Cana        CGAGTGTTTGGGTGTAAAACCCGTACGCGTAATGAAAGTGAACGAAGGTGGGGGCCCA--
Cang        CGAGTGTTTGGGTGTTAAACCCGTACGCGTAATGAAAGTGAACGTAGGTTGGGGCCCTCC
Sacc        CGAGTGTTTGGGTGTAAAACCCATACGCGTAATGAAAGTGAACGTAGGTTGGGGCCTC--
Malg        CGAGTTTTTGGGTGGCAAACCCATGAGCGTAATGAAAGTGAATACAGGTGGGATCCGC--
Cryn        CGAGTGTTTGAGTGTCAAACTCGAGCGCGAAATGAAAGTGAATGTAGGAGGGATCCGC--
Cutd        CGAGTGTTTGAGTGTCAAACTCGAGCGCGTAATGAAAGTGAAC-TGGGAGGGATCCGT--
            ***** ** * ***  **** *    *** ****** ***    **   *  *

Fusk        --------GGCGCATCATCGACCGATCCTGATGTTATCGGATGGATTTGAGTAAGAGCAT
Penc        ----CACGGGTGCACCATCGACCGATCCTGAAGTCTTCGGATGGATTTGAGTAAGAGCGT
Aspf        -CTCGCGGGGCGCACCATCGACCGATCCTGATGTCTTCGGATGGATTTGAGTACGAGCGT
Aspn        -CTTGCGGGGCGCACCATCGACCGATCCTGATGTCTTCGGATGGATTTGAGTAAGAGCGT
```

```
Cana            ----TTAGGGTGCACCATCGACCGATCCTGATGTGTTCGGATGGATTTGAGTAAGAGCAT
Cang            ACCTGGGGGGTGCACAATCGACCGATCCTGATGTCTTCGGATGGATTTGAGTAAGAGCAT
Sacc            -GCA-AGAGGTGCACAATCGACCGATCCTGATGTCTTCGGATGGATTTGAGTAAGAGCAT
Malg            ------AAGGAGCACCATCGACCGGCCCTGAACTTCTGGGATGGGTCTGAGTAAGAGCAT
Cryn            ------AAGGAGCACCTTCGACCGATCCGGATCTTCTGTGATGGATTTGAGTAAGAGCAT
Cutd            ------AAGGAGCACCTTCGACCGATCCGGATGTTTACTGATGGATTTGAGTAAGAGCAT
                        ** ***   *******  ** **  *     ***** * ****** **** *

Fusk            ACGGGGCCGGACCCGAAAGAAGGTGAACTATGCCTGTGTAGGGTGAAGCCAGAGGAAACT
Penc            AGCTGTTGGGACCCGAAAGATGGTGAACTATGCCTGAATAGGGCGAAGCCAGAGGAAACT
Aspf            AGCTGTGGGGACCCGAAAGATGGTGAACTATGCCTGAATAGGGCGAAGCCAGAGGAAACT
Aspn            AGCTGTGGGGACCCGAAAGATGGTGAACTATGCCTGAATAGGGCGAAGCCAGAGGAAACT
Cana            AGCTGTTGGGACCCGAAAGATGGTGAACTATGCCTGAATAGGGTGAAGCCAGAGGAAACT
Cang            AGCTGTTGGGACCCGAAAGATGGTGAACTATGCCTGAATAGGGTGAAGCCAGAGGAAACT
Sacc            AGCTGTTGGGACCCGAAAGATGGTGAACTATGCCTGAATAGGGTGAAGCCAGAGGAAACT
Malg            ATATGTTGGGACCCGAAAGATGGTGAACTATGCCTGAATAGGGCGAAGCCAGAGGAAACT
Cryn            ATATGCTGGGACCCGAAAGATGGTGAACTATGCCTGAATAGGGCGAAGCCAGGGGAAACT
Cutd            ATATGCTGGGACCCGAAAGATGGTGAACTATGCCTGAATAGGGCGAAGCCAGGGGAAACT
                *   *   ************ ***************  ***** ******** *******

Fusk            CTGGTGGAGGCTCGCAGCGGTTCTGACGTGCAAATCGATCGTCAAACATGGGCATGGGGG
Penc            CTGGTGGAGGCTCGTAGCGGTTCTGACGTGCAAATCGATCGTCGAATTTGGGTAT-AGGG
Aspf            CTGGTGGAGGCTCGCAGCGGTTCTGACGTGCAAATCGATCGTCAAATTTGGGTAT-AGGG
Aspn            CTGGTGGAGGCTCGCAGCGGTTCTGACGTGCAAATCGATCGTCAAATTTGGGTAT-AGGG
Cana            CTGGTGGAGGCTCGTAGCGGTTCTGACGTGCAAATCGATCGTCGAATTTGGGTAT-AGGG
Cang            CTGGTGGAGGCTCGTAGCGGTTCTGACGTGCAAATCGATCGTCGAATTTGGGTAT-AGGG
Sacc            CTGGTGGAGGCTCGTAGCGGTTCTGACGTGCAAATCGATCGTCGAATTTGGGTAT-AGGG
Malg            CTGGTGGAGGCTCGCAGCGGTTCTGACGTGCAAATCGATCGTCAAATTTGGGTAT-AGGG
Cryn            CTGGTGGAGGCTCGTAGCGATTCTGACGTGCAAATCGATCGTCGAATTTGGGTAT-AGGG
Cutd            CTGGTGGAGGCTCGTAGCGATTCTGACGTGCAAATCGATCGTCAAATTTGGGTAT-AGGG
                ************** **** *********************** **  **** **  ***
                                                          LR5 primer
Fusk            GCGAAAGACTAATCGAACCTTCTAGTAGCTGGTTTCCGCCGAAGTTTCCCTCAGGATAGC
Penc            GCGAAAGACTAATCGAACCATCTGGTAGCTGGTTCCTGCCGAAGTTTCCCTCAGGATAGC
Aspf            GCGAAAGACTAATCGAACCATCTAGTAGCTGGTTCCTGCCGAAGTTTCCCTCAGGATAGC
Aspn            GCGAAAGACTAATCGAACCATCTAGTAGCTGGTTCCTGCCGAAGTTTCCCTCAGGATAGC
Cana            GCGAAAGACTAATCGAACCATCTAGTAGCTGGTTCCTGCCGAAGTTTCCCTCAGGATAGC
Cang            GCGAAAGACTAATCGAACCATCTAGTAGCTGGTTCCTGCCGAAGTTTCCCTCAGGATAGC
Sacc            GCGAAAGACTAATCGAACCATCTAGTAGCTGGTTCCTGCCGAAGTTTCCCTCAGGATAGC
Malg            GCGAAAGACTAATCGAACCATCTAGTAGCTGGTTCCTGCCGAAGTTTCCCTCAGGATAGC
Cryn            GCGAAAGACTAATCGAACCATCTAGTAGCTGGTTCCTGCCGAAGTTTCCCTCAGGATAGC
Cutd            GCGAAAGACTAATCGAACCATCTAGTAGCTGGTTCCTGCCGAAGTTTCCCTCAGGATAGC
                ******************* *** ********** * ***********************

Fusk            AGTG--TTGAACTCAGTTTTATGAGGTAAAGCGAATGATTAGGGGACTCGGGGGGCGCTA
Penc            AGTAACGCGAATTCAGTTTTATGAGGTAAAGCGAATGATTAGAGGCCTTGGGG---TTGA
Aspf            AGTAACGC-GGATCAGTTTTATGAGGTAAAGCGAATGATTAGAGGCCTTGGGG---TTGA
Aspn            AGTAACGCAAAATCAGTTTTATGAGGTAAAGCGAATGATTAGAGGCATTGGGG---TTGA
Cana            AGAAGCTC-GTATCAGTTTTATGAGGTAAAGCGAATGATTAGAAGTCTTGGGG---TTGA
Cang            AGAAGCTC-GTATCAGTTTTATGAGGTAAAGCGAATGATTAGAGGTACCGGGG---TTGA
Sacc            AGAAGCTC-GTATCAGTTTTATGAGGTAAAGCGAATGATTAGAGGTTCCGGGG---TCGA
Malg            AGAAACTC-GTATCAGTTTTATGAGGTAAAGCGAATGATTAGAGGCCTTGGGG---ATGT
Cryn            AGAAACTC-GCATCAGTTTTATGAGGTAAAGCGAATGATTAGAGGCCTTGGGG---ACGA
Cutd            AGAAACTC-GCATCAGTTTTATGAGGTAAAGCGAATGATTAGAGGCCTTGGGG---ACGA
                **          ******************************  *    ****

Fusk            TTTAGCCTTCATCCATTCTCAAACTTTAAATATGTAAGAAGCCCTTGTTGCTTAATTGAA
Penc            AACAACCTTAACCTATTCTCAAACTTTAAATATGTAAGAAGCCCTTGTTGCTTAATTGAA
Aspf            AACAACCTTAACCTATTCTCAAACTTTAAATATGTAAGAAGCGCTTGTTGCTTAGTTGAA
Aspn            AACAACCTTAACCTATTCTCAAACTTTAAATATGTAAGAAGCCCTTGTTGCTTAGTTGAA
Cana            AATGACCTTAACTTATTCTCAAACTTTAAATATGTAAGAAGTCCTTGTTGCTTAATTGAA
Cang            AATGACCTTGACCTATTCTCAAACTTTAAATATGTAAGAAGTCCTTGTTGCTTAATTGAA
Sacc            AATGACCTTGACCTATTCTCAAACTTTAAATATGTAAGAAGTCCTTGTTACTTAATTGAA
Malg            AACATCCTTAACCTATTCTCAAACTTTAAATGTGTAAGAAGTCCTTGTTACTTAAGTGAA
Cryn            AACGTCCTTAACCTATTCTCAAACTTTAAATGTGTAAGAAGCACTTGTCACTTAATTGGA
Cutd            AACGTCCTTAACCTATTCTCAAACTTTAAATATGTAAGAAGCACTTGTCACTTAATTGGA
                     **** *   ***************** *********  *****  ****  ** *

Fusk            CGTGGGCATTCGAATGAATCAACACTAGTGGGCCATTTTTTGGTAAGCAGAACTGGCGAT
Penc            CGTGGGCATTAGAATGATGCGTTACTAGTGGGCCA-TTTTTGGTAAGCAGAACTGGCGAT
Aspf            CGTGCGCATTAGAATGAAGCGTTACTAGTGGGCCA-TTTTTGGTAAGCAGAACTGGCGAT
Aspn            CGTGGGCATTAGAATGGAGCGTTACTAGTGGGCCA-TTTTTGGTAAGCAGAACTGGCGAT
Cana            CGTGGACAATTGAATGAAGAGCTTTTAGTGGGCCA-TTTTTGGTAAGCAGAACTGGCGAT
Cang            CGTGGACATTTGAATGAAGAGCTTTTAGTGGGCCA-TTTTTGGTAAGCAGAACTGGCGAT
Sacc            CGTGGACATTTGAATGAAGAGCTTTTAGTGGGCCA-TTTTTGGTAAGCAGAACTGGCGAT
Malg            CGTGGACATGCGAATGA-GAGTTTCTAGTGGGCCA-TTTTTGGTAAGCAGAACTGGCGAT
Cryn            CGAGCGCATGCGAATGA-GAGTTTCTAGTGGGCCA-TTTTTGGTAAGCAGAACTGGCGAT
Cutd            CGTGTGCATGCGAATGA-GAGTTTCTAGTGGGCCA-TTTTTGGTAAGCAGAACTGGCGAT
                ** *  **   *****         ********** ************************

Fusk            GCGGGATGAACCGAACGCGAGGTTAAGGTGCCAGAGTAGACGCTCATCAGACACCAAAAA
Penc            GCGGGATGAACCGAACGCGAGGTTAAGGTGCCGGAATACACGCTCATCAGACACCACAAA
Aspf            GCGGGATGAACCGAACGCGAGGTTAAGGTGCCGGAATGCACGCTCATCAGACACCACAAA
Aspn            GCGGGATGAACCGAACGCGAGGTTAAGGTGCCGGAATGCACGCTCATCAGACACCACAAA
Cana            GCGGGATGAACCGAACGTGAAGTTAAAGTGCCGGAATGCACGCTCATCAGACACCACAAA
Cang            GCGGGATGAACCGAACGTGGAGTTAAGGTGCCGGAATACACGCTCATCAGACACCACAAA
Sacc            GCGGGATGAACCGAACGTAGAGTTAAGGTGCCGGAATACACGCTCATCAGACACCACAAA
Malg            GCGGGATGAACCGATCGTGAGGTTAAGGTGCCGGAATACACGCTCATCAGACACCACAAA
Cryn            GCGGGATGAACCGATCGTGAGGTTAAGGTGCCGGAATACACGCTCATCAGACACCACAAA
Cutd            GCGGGATGAACCGATCGTGAGGTTAAGGTGCCGGAATACACGCTCATCAGACCCCACAAA
                ************** **    ***** ***** ** *  ************* *** ***

Fusk            AGGTGTTAGTACATCTTGACAGCAGGACGGTGGCCATGGAAGTCGGAATCCGCTAAGGAC
Penc            AGGTGTTAGTTCATCTAGACAGCCCGACGGTGGCCATGGAAGTCGGAATCCGCTAAGGAG
Aspf            AGGTGTTAGTTCATCTAGACAGCCCGACGGTGGCCATGGAAGTCGGAATCCGCTAAGGAG
Aspn            AGGTGTTAGTTCATCTAGACAGCCCGACGGTGGCCATGGAAGTCGGAATCCGCTAAGGAG
Cana            AGGTGTTAGTTCATCTAGACAGCCGGACGGTGGCCATGGAAGTCGGAATCCGCTAAGGAG
Cang            AGGTGTTAGTTCATCTAGACAGCCGGACGGTGGCCATGGAAGTCGGAATCCGCTAAGGAG
Sacc            AGGTGTTAGTTCATCTAGACAGCCGGACGGTGGCCATGGAAGTCGGAATCCGCTAAGGAG
Malg            AGGTGTTAGTTCATCTAGACAGCAGGACGGTGGCCATGGAAGTTGGAATCCGCTAAGGAG
Cryn            AGGTGTTAGTTCATCTAGACAGCAGGACGGTGGCCATGGAAGTCGGAATCCGCTAAGGAG
Cutd            AGGTGTTAGTTCATCTAGACAGCAGGACGGTGGCCATGGAAGTCGGAATCCGCTAAGGAG
                ********** ***** ******  ****************** ***************

Fusk            TGTGTAACAACTCACCTGCCGAATGTACTAGCCCTGAAAATGGATGGCGCTCAAGCGTCT
Penc            TGTGTAACAACTCACGGGCCGAATGAACTAGCCCTGAAAATGGATGGCGCTTAAGCGTGT
Aspf            TGTGTAACAACTCACGGGCCGAATGAACTAGCCCTGAAAATGGATGGCGCTCAAGCGTGC
Aspn            TGTGTAACAACTCACGGGCCGAATGAACTAGCCCTGAAAATGGATGGCGCTCAAGCGTGC
Cana            TGTGTAACAACTCACCGGCCGAATGAACTAGCCCTGAAAATGGATGGCGCTCAAGCGTGC
Cang            TGTGTAACAACTCACCGGCCGAATGAACTAGCCCTGAAAATGGATGGCGCTCAAGCGTGT
Sacc            TGTGTAACAACTCACCGGCCGAATGAACTAGCCCTGAAAATGGATGGCGCTCAAGCGTGT
Malg            TGTGTAACAACTCACCTGCCGAATGAACTAGCCCTGAAAATGGATGGCGCTTAAGCGTGT
Cryn            TGTGTAACAACTCACCTGCCGAATGAACTAGCCCTGAAAATGGATGGCGCTCAAGCGTGT
Cutd            TGTGTAACAACTCACCTGCCGAATGAACTAGCCCTGAAAATGGATGGCGCTCAAGCGTGT
                ***************  ******** ************************* ******

Fusk            CACCCATACCTCGCCCTCAGGGTAGAAACGATGCCCTGAGGAGTAGGCGGACGTGGAGGT
Penc            TACCCATACCTCGCCGTCAGGGTAGAAACGATGCCCTGACGAGTAGGCAGGCGTGGGGGT
Aspf            TACCCATACCTCGCCGTCGGGGTAGAAACGACGCCCCGACGAGTAGGCAGGCGTGGGGGT
Aspn            TACCCATACCTCGCCGTCGGGGTAGAAACGATGCCCCGACGAGTAGGCAGGCGTGGGGGT
Cana            TACTTATACTTCACCGTGATTGCTGTTTTGACGCTTTCACGAGTAGGCAGGCGTGGAGGT
Cang            TACCTATACTCCGCCGTCAGGGTTGAAATGAGGCCCTGACGAGTAGGCAGGCGTGGGGGT
Sacc            TACCTATACTCTACCGTCAGGGTTGATATGATGCCCTGACGAGTAGGCAGGCGTGGAGGT
Malg            TACCTATACCTCACCGTCAATGTTAGAGCGATGCGTTGACGAGTAGGCAGGCGTGGAGGT
Cryn            TACCCATACCTCACCGTCAGCGTTGTAGTGACGCGCTGACGAGTAGGCAGGCGTGGAGGT
Cutd            TACCCATACCTCACCGTCAGCGTTTAAGTGACGCGCTGACGAGTAGGCAGGCGTGGAGGT
```

```
                 **  ****    ** *    *       ** **    * ******** * ***** ***
                                                                  LR7 primer
Fusk            CAGTGACGAAGCCTAGGGCGTGAGCCCGGGTTGAACGGCCTCTAGTGCAGATCTTGGTGG
Penc            CCGTGACGAAGCCTTGGGAGTGATCCCGGGTCGAACGGCCCCTAGTGCAGATCTTGGTGG
Aspf            CCGTGACGAAGCCTTGGGAGTGATCCCGGGTCGAACGGCCCCTAGTGCAGATCTTGGTGG
Aspn            CCGTGACGAAGCCTTGGGAGTGATCCCGGGTCGAACGGCCCCTAGTGCAGATCTTGGTGG
Cana            CAGTGACGAAGCCTTTGCTGTAAAGCTGGGTCGAACGGCCTCTAGTGCAGATCTTGGTGG
Cang            CAGTGACGAAGCCTAGGCCGTAAGGTCGGGTCGAACGGCCCCTAGTGCAGATCTTGGTGG
Sacc            CAGTGACGAAGCCTAGACCGTAAGGTCGGGTCGAACGGCCTCTAGTGCAGATCTTGGTGG
Malg            T-GTATCGAAGCCTAGGCAGTGATGCTGGGTGGAACAGCCTCTAGTGCAGATCTTGGTGG
Cryn            CAGTGTAGAAGCCTAGGCAGTGATGTCGGGTGGAACGGCCTCTAGTGCAGATCTTGGTGG
Cutd            CAGTGAAGAAGCCTTGGCAGTGATGCTGGGTGAAACGGCCTCTAGTGCAGATCTTGGTGG
                  **   *******     ** *    ****  *** *** *******************

Fusk            TAGTAGCAAATACTTCAATGAGAACTTGAAGGACCGAAGTGGGGAAAGGTTCCATGTGAA
Penc            TAGTAGCAAATACTCAAATGAGAACTTTGAGGACTGAAGTGGGGAAAGGTTCCATGTGAA
Aspf            TAGTAGCAAATACTCAAATGAGAACTTTGAGGACTGAAGTGGGGAAAGGTTCCATGTGAA
Aspn            TAGTAGCAAATACTCAAATGAGAACTTTGAGGACTGAAGTGGGGAAAGGTTCCATGTGAA
Cana            TAGTAGCAAATATTCAAATGAGAACTTTGAAGACTGAAGTGGGGAAAGGTTCCATGTCAA
Cang            TAGTAGCAAATATTCAAATGAGAACTTTGAAGACTGAAGTGGGGAAAGGTTCCACGTCAA
Sacc            TAGTAGCAAATATTCAAATGAGAACTTTGAAGACTGAAGTGGGGAAAGGTTCCACGTCAA
Malg            TAGTAGCAAATATTCAAGTGAGAACCTTGAAGACTGAAGTGGAGAAAGGTTCCATGGTAA
Cryn            TAGTAGCAAATATTCAAGTGAGAACCTTGAAGACTGAAGTGGAGAAAGGTTCCATGGTAA
Cutd            TAGTAGCAAATATTCAAGTGAGAACCTTGAAGACTGAAGTGGAGAAAGGTTCCATGGTAA
                ************ *  * ******* *  * *** ******* *********** *  **

Fusk            CAGCGGTTGGACATGGGTTAGTCGATCCTAAGCCATAGGGAAGTTCCGTTTCAAAGGCGC
Penc            CAGCAGTTGGACATGGGTTAGTCGATCCTAAGGCATAGGGAAGTTCCGTTTGAAAGGCGC
Aspf            CAGCAGTTGGACATGGGTTAGTCGATCCTAAGGCATAGGGAAGTTCCGTTTGAAAGGCGC
Aspn            CAGCAGTTGGACATGGGTTAGTCGATCCTAAGGCATAGGGAAGTTCCGTTTGAAAGGCGC
Cana            CAGCAGTTGGACATGGGTTAGTCGATCCTAAGAGATGGGGAAGCTCCGTTTCAACG-TGC
Cang            CAGCAGTTGGACGTGGGTTAGTCGATCCTAAGAGATGGGGAAGCTCCGTTTCAAAG--GC
Sacc            CAGCAGTTGGACGTGGGTTAGTCGATCCTAAGAGATGGGGAAGCTCCGTTTCAAAG--GC
Malg            CAGCAGTTGGACATGGGTGAGCCGATCCTAAGAGATAGGGTAGTTCCGTATGAAAGTTGC
Cryn            CAGCAGTTGGACATGGGTCAGTCGATCCTAAGAGATAGGGAAACTCCGTTTTAAAG-CGC
Cutd            CAGCAGTTGGACATGGGTTAGTCGATCCTAAGAGATAGGGAAGCTCCGTTTTAAAG-TAC
                **** ******* ***** ** **********  ** *** *  ***** * ** *   *

Fusk            AC-------TATGCGCCGTCTGGCGAAAGGGGAGCCGGTCAATATTCCGGCACCTGGATG
Penc            CCT------CGTGCGCCGTGTGCCGAAAGGGAAGCCGGTTAACATTCCGGCACCTAGATG
Aspf            CCT------CGTGCGCCGTGTGCCGAAAGGGAAGCCGGTTAACATTCCGGCACCTGGATG
Aspn            CCT------CGTGCGCCGTGTGCCGAAAGGGAAGCCGGTTAACATTCCGGCACCTGGATG
Cana            TTGATTT--TTCAGGCCAACCATCGAAAGGGAATCCGGTTAAAATTCCGGAACTTGGATA
Cang            CTGATTTA-TGCAGGCCACC-ATCGAAAGGGAATCCGGTTAAGATTCCGGAACCTGGATG
Sacc            CTGATTTTATGCAGGCCACC-ATCGAAAGGGAATCCGGTTAAGATTCCGGAACCTGGATA
Malg            GCGATTC--TTCGCGCCGCCTATCGAAAGGGAAGCCGGTTAAAATTCCGGCGCCAGGATA
Cryn            ACGATTT--TCCGTGCCGCCTATCGAAAGGGAATCCGGTTAAGATTCCGGAACCAGGATG
Cutd            ACGATTT--TCCGTGTCGCCTATCGAAAGGGAATCCGGTTAAGATTCCGGAACCAGGATG
                              * *      ******** * ***** ** *******  *   ***

Fusk            TGGGTTTTGCGCGGCAACGCAACTGAACGCGGAGACGACGGCGGGGGCCCCGGGCAGAGT
Penc            TGGATTCTCCACGGCAACGTAACTGAACGCGGAGACGTCGGCGGGGGTCCTGGGAAGAGT
Aspf            TGGATTCTCCACGGCAACGTAACTGAACGCGGAGACGTCGGCGGGGGTCCTGGGAAGAGT
Aspn            TGGATTCTCCACGGCAACGTAACTGAACGCGGAGACATCGGCGGGGGTCCTGGGAAGAGT
Cana            TGGATTCTTCACGGCAACGTAACTGAATGTGGAGACGTCGGCGTGAGCCCTGGGAGGAGT
Cang            TGGATTCTTCACGGCAACGTAACTGAATGTGGAGACGTCGGCGCGAGCCCTGGGAGGAGT
Sacc            TGGATTCTTCACGGTAACGTAACTGAATGTGGAGACGTCGGCGCGAGCCCTGGGAGGAGT
Malg            TGGATGTTAGACGGCAACGTAAACGAACCAGGAGACGTCGGCGAGGGCCCTGGAAAGAGT
Cryn            TGGATCATTGACGGTAACGTAAATGAAGTTGGAGACGTCGGCAAGGGCCCTGGGAAGAGT
Cutd            TGGATCTTTAACGGCAACGTAAATGAAGTTGGAGACGCTGGCAAGGGCCCTGGGAAGAGT
                *** *  *   *** **** **  ***   ******   ***  * * ** **   ****

Fusk            TCTCTTTTTCTTCTTAACAGTCTCTCACCCTGAAATCGGTTTGTCCGGAGCTAGGGTTTA
Penc            TCTC-TTTTCTTCTTGACAGCCTATCACCCTGAAATCGGTTTGTCCGGAGCTAGGGTTCT
Aspf            TCTC-TTTTCTTCTTGACAGCCTTCCACCCTGAAATCGGTTTGTCCGGAGCTAGGGTTCC
Aspn            TCTC-TTTTCTTCTTGACGGCCTATCACCCTGAAATCGGTTTGTCCGGAGCTAGGGTTCC
Cana            TATC-TTTTCTTCTTAACAGCTTATCACCCTGGAATTGGTTTATCCGGAGATGGGGTCTT
Cang            TATC-TTTTCTTCTTAACAGCTTATCACCCTGGAATTGGTTTATCCGGAGATGGGGTCTT
Sacc            TATC-TTTTCTTCTTAACAGCTTATCACCCCGGAATTGGTTTATCCGGAGATGGGGTCTT
Malg            TCTC-TTTTCTTTCTTACAGTCTACCACCCTGAAATCGATTTATTCGGAGCTAGGGTTCT
Cryn            TCTC-TTTTCTCCTTAACCGCCTACGACCTCGAAATCGGATTATCCGGAGCTGAGGTTAT
Cutd            TCTC-TTTTCTCCTTAACCGCTTACGACCTTGAAATCGGATTATCCGGAGCTAAGGTTAT
                * ** ******   * ** *  *   ***  * *** *  ** * ***** *  ***

Fusk            ATGGCTGGAAGAGCCCAGCACCTCTGCTGGGTCCGGTGCGCTCTCGACGTCCCTTGAAAA
Penc            ATGGCTGGCAGAGCGCCGCACTTTTGCGGCGTCCGGTGCGCCCCCGACGACCCTTGAAAA
Aspf            ATGGCTGGCAGAGCCCCGCACCTTTGCGGGGTCCGGTGCGCCCCCGACGACCCTTGAAAA
Aspn            ACGGCCGGCAGAGCCCTGCACCTTTGCAGGGTCCGGTGCGCCCCCGACGATCCTTGAAAA
Cana            ATGGCTGGAAGAGCGCGGTAATTTTGCCGCGTCCGGTGCGCTTACGACGGTCCTTGAAAA
Cang            ATGGCTGGAAGAGGCGAGCTCATATGCTCGCTCCGGTGCGCTTGCGACGGCCCTTGAAAA
Sacc            ATGGCTGGAAGAGGCCAGCACCTTTGCTGGCTCCGGTGCGCTTGTGACGGCCCGTGAAAA
Malg            ATGGCTGGTAGAGCTCGGCACTTCTGCCGAGTCCGGTGCGTCCTCGACGGCCCTTGAAAA
Cryn            ATGGTGGGTAAAGCACAACACCTCTGTTGTGTCCGGTGCGTCCTTGACGATCCTTGAAAA
Cutd            ATGGCGGGTAGAGCACAACACCTCTGTTGTGTCCGGTGCGTCCTTGACAGTCCTTGAAAA
                * **  ** * **         * **     *********     ***   ** ******

Fusk            TCCGCGGGAAGAAATAATTCTCACGCCAGGTCGTACTCATAACCGCAGCAGGTCTCCAAG
Penc            TCCGCGGGAAGGAATAGTTTTCACGCTAGGTCGTACTCATAACCGCAGCAGGTCTCCAAG
Aspf            TCCGCGGGAAGGAATAGTTTTCACGCCAGGTCGTACTCATAACCGCAGCAGGTCTCCAAG
Aspn            TCCGCGGGAAGGAATAGTTTTCACGCCAGGTCGTACTCATAACCGCAGCAGGTCTCCAAG
Cana            TCCACAGGAAGGAATAGTTTTCATGCCAAGTCGTACTCATAACCGCAGCAGGTCTCCAAG
Cang            TCCACAGGAAGGAATAGTTTTCACGCCAGGTCGTACTGATAACCGCAGCAGGTCTCCAAG
Sacc            TCCACAGGAAGGAATAGTTTTCATGCCAGGTCGTACTGATAACCGCAGCAGGTCTCCAAG
Malg            TCC-TGGGGAGCATTCCATCTCATGCCTGGTCGTACTGATAACCGCAGCAGGTCTCCTAG
Cryn            TCCGACGGAACGTATAAGTCTCACGCCTGGTCGTACTCATAACCGCAGCAGGTCTCCAAG
Cutd            TCCGACGGAATGTATAAGTCTCACGCCTGGTCGTACTCATAACCGCAGCAGGTCTCCAAG
                ***   ** *    *   * *** **   ******** ******************* **

Fusk            GTGAACAGCCTCTGGTTGATAGAACAATGTAGATAAGGGAAGTCGGCAAAATAGATCCGT
Penc            GTGAACAGCCTCTAGTTGATAGAACAATGTAGATAAGGGAAGTCGGCAAAATGGATCCGT
Aspf            GTGAACAGCCTCTAGTTGATAGAACAATGTAGATAAGGGAAGTCGGCAAAATGGATCCGT
Aspn            GTGAACAGCCTCTAGTTGATAGAACAATGTAGATAAGGGAAGTCGGCAAAATGGATCCGT
Cana            GTTAACAGCCTCTAGTTGATAGAATAATGTAGATAAGGGAAGTCGGCAAAATAGATCCGT
Cang            GTGAACAGCCTCTAGTTGATAGAATAATGTAGATAAGGGAAGTCGGCAAAATAGATCCGT
Sacc            GTGAACAGCCTCTAGTTGATAGAATAATGTAGATAAGGGAAGTCGGCAAAATAGATCCGT
Malg            GTTAACAGCCTCTAGTTGATAGAATAATGTGGATAAGGGAATTCGGCAAATTGGATCCGT
Cryn            GTGAACAGCCTCTAGTTGATGGAACAATGTAGATAAGGGAAGTCGGCAAAATAGATCCGT
Cutd            GTGAACAGCCTCTAGTTGATAGAACAATGTAGATAAGGGAAGTCGGCAAAATAGATCCGT
                ** ********** ****** *** ***** ********** ******** * *******

Fusk            AACTTCGGGATAAGGATTGGCTCTAAGGGTTGGGCACGCAGGGCCTTGGGCGGACGCCAT
Penc            AACTTCGGGATAAGGATTGGCTCTAAGGGTCGGGCTCGCTGGGCCTTGGGGGGAACC-TC
Aspf            AACTTCGGGATAAGGATTGGCTCTAAGGGTCGGGCCCGCTGGGCCTTGGGGGAAACC-CC
Aspn            AACTTCGGGATAAGGATTGGCTCTAAGGGTCGGGCTCGCTGGGCCTTGGGGGAAACC-CC
Cana            AACTTCGGGATAAGGATTGGCTCTAAGGATCGGGTGTCTTGGGCCTTGTGTAGACGC---
Cang            AACTTCGGGATAAGGATTGGCTCTAAGGGTCGGGTAGTGAGGGCCTTGGTCAGACGC-GG
Sacc            AACTTCGGGATAAGGATTGGCTCTAAGGGTCGGGTAGTGAGGGCCTTGGTCAGACGC-AG
Malg            AACTTCGGGATAAGGATTGGCTCTAAGGGTTGGGTACATCGGGCCCTGGTTGGAAGC-CG
Cryn            AACTTCGGGATAAGGATTGGCTCTAAGGGTTGGGTGCGTCGGGCCGTTGACGGAAGGAAG
Cutd            AACTTCGGGATAAGGATTGGCTCTAAGGGTTGGGTATGTCGGGCCTTGAGTAGAAGTGAG
                **************************** * ***      ***** *      *

Fusk            GGGGGGCAGGCTGCTTTTA--GCCGGGCAACCGGC---------CGGCGGCGGCCAGCAC
Penc            CTGGAGCAGTAGGGCACTA--GCCGGGCAACCGGC---------CGGCGCCCCGCAGCAC
Aspf            TCGGAGCAGGGGGGCACTA--GCCGGGCAACCGGC---------CGGCGCCCCCCAGCAC
Aspn            TCGGAGCAGGGGGGCACTA--GCCGGGCAACCGGC---------CGGCGCCCCCCAGCAC
```

```
Cana            --------GGCGGTGACTGTTGGCGGGCTGTTTTA------CGACGGACTGCTGGTGGAT
Cang            CGGGGCTGCGTGCGGACTGC-CTGGTGGGGCTTGCTCTGCCGGGCGGACTGCATGCGGCT
Sacc            CGGGCGTGCTTGTGGACTGC-TTGGTGGGGCTTGCTCTGCTAGGCGGACTACTTGCGTGC
Malg            CTGATGCTGGCTTGGACTGCTGCGTGGCAACATGC-------GGTGGACCGAGCCGGCGT
Cryn            CTGGACCTGGCG-GGACTGC-ATGGGGCAACCTGT--------GTGGACCT-GCTGGGAT
Cutd            CTGGACTAGGCT-GGACTGA-GTGGGGCAACCTAC--------TCGGACCG-GCCCGGAC
                                 *        *                  **         *

Fusk            CCGTGCGCTGATGCCCTTGGCAGGCTTC-----GGCCGTC-CGGCGTGCGGTTAACAACC
Penc            CGGGTTGGGGACGCCCTTGGCAGGCTTC-----GGCCGTC-CGGCGGGCGATTAACGACC
Aspf            TGGGGCGGGGACGCCCTTGGCAGGCTTC-----GGCCGTC-CGGCGGGCGCTTAACGACC
Aspn            CGGGTGGGGGACGCCCTTGGCAGGCTTC-----GGCCGTC-CGGCGGGCGCTTAACGACC
Cana            GCTGCTGTAGACACGCTTGGTAGGTCTTTAT--GGCCGTC-CGGGGCACGTTTAACGATC
Cang            CCTGTCGTAGACGGTCTTGGTAGGTCTCTTGTAGGCCGTCGCTTGCTGCGATTAACGATC
Sacc            CTTGTTGTAGACGGCCTTGGTAGGTCTCTTGTAGACCGTCGCTTGCTACAATTAACGATC
Malg            CGGCGCGTGGACGGCCAGGGCAGTCCCTCG---GGACGGC-CGGTGTACGCTTAACAACC
Cryn            CGGCGACTGGAAGTCTTTGGCAGCCCTC-----GGGCGTC-CGGCGTACGCTTAACAACC
Cutd            TGGCGACCGGAAGCTCTTGGCAGTCTTC-----GGGCCTC-CGGCATACAATTAACAACC
                         **       ** **          *  *  * *      *  ***** * *
                                                  RCA95m primer
Fusk            AACTTAGAACTGGTACGGACAAGGGGAATCTGACTGTCTAATTAAAACATAGCATTGCGA
Penc            AACTTAGAACTGGTACGGACAAGGGGAATCTGACTGTCTAATTAAAACATAGCATTGCGA
Aspf            AACTTAGAACTGGTACGGACAAGGGGAATCTGACTGTCTAATTAAAACATAGCATTGCGA
Aspn            AACTTAGAACTGGTACGGACAAGGGGAATCTGACTGTCTAATTAAAACATAGCATTGCGA
Cana            AACTTAGAACTGGTACGGACAAGGGGAATCTGACTGTCTAATTAAAACATAGCATTGTGA
Cang            AACTTAGAACTGGTACGGACAAGGGGAATCTGACTGTCTAATTAAAACATAGCATTGCGA
Sacc            AACTTAGAACTGGTACGGACAAGGGGAATCTGACTGTCTAATTAAAACATAGCATTGCGA
Malg            AGCTTAGAACTGGTACGGACAAGGGGAATCTGACTGTCTAATTAAAACATAGCATTGCGA
Cryn            AACTTAGAACTGGTACGGACAAGGGGAATCTGACTGTCTAATTAAAACATAGCATTGCGA
Cutd            GACTTAGAACTGGTACGGACAAGGGGAATCTGACTGTCTAATTAAAACATAGCATTGCGA
                  ******************************************************* **

Fusk            TGGCCAGAAAGTGGTGTTGACGCAATGTGATTTCTGCCCAGTGCTCTGAATGTCAAAGTG
Penc            TGGCCAGAAAGTGGTGTTGACGCAATGTGATTTCTGCCCAGTGCTCTGAATGTCAAAGTG
Aspf            TGGCCAGAAAGTGGTGTTGACGCAATGTGATTTCTGCCCAGTGCTCTGAATGTCAAAGTG
Aspn            TGGCCAGAAAGTGGTGTTGACGCAATGTGATTTCTGCCCAGTGCTCTGAATGTCAAAGTG
Cana            TGGTCAGAAAGTGATGTTGACACAATGTGATTTCTGCCCAGTGCTCTGAATGTCAAAGTG
Cang            TGGTCAGAAAGTGATGTTGACGCAATGTGATTTCTGCCCAGTGCTCTGAATGTCAAAGTG
Sacc            TGGTCAGAAAGTGATGTTGACGCAATGTGATTTCTGCCCAGTGCTCTGAATGTCAAAGTG
Malg            TGGCCAGAAAGTGGTGTTGACGCAATGTGATTTCTGCCCAGTGCTCTGAATGTCAAAGTG
Cryn            TGGCCAGAAAATGGTGTTGACGCAATGTGATTTCTGCCCAGTGCTCTGAATGTCAAAGTG
Cutd            TGGCCAGAAAGTGGTGTTGACGCAATGTGATTTCTGCCCAGTGCTCTGAATGTCAAAGTG
                *** ****** ** ******* **************************************

Fusk            AAGTAATTCAACCAAGCGCGGGTAAACGGCGGGAGTAACTATGACTCTCTTAAGGTAGCC
Penc            AAGAAATTCAACCAAGCGCGGGTAAACGGCGGGAGTAACTATGACTCTCTTAAGGTAGCC
Aspf            AAGAAATTCAACCAAGCGCGGGTAAACGGCGGGAGTAACTATGACTCTCTTAAGGTAGCC
Aspn            AAGAAATTCAACCAAGCGCGGGTAAACGGCGGGAGTAACTATGACTCTCTTAAGGTAGCC
Cana            AAGAAATTCAACCAAGCGCGGGTAAACGGCGGGAGTAACTATGACTCTCTTAAGGTAGCC
Cang            AAGAAATTCAACCAAGCGCGGGTAAACGGCGGGAGTAACTATGACTCTCTTAAGGTAGCC
Sacc            AAGAAATTCAACCAAGCGCGGGTAAACGGCGGGAGTAACTATGACTCTCTTAAGGTAGCC
Malg            AAGAAATTCAACCAAGCGCGGGTAAACGGCGGGAGTAACTATGACTCTCTTAAGGTAGCC
Cryn            AAGAAATTCAACCAAGCGCGGGTAAACGGCGGGAGTAACTATGACTCTCTTAAGGTAGCC
Cutd            AAGAAATTCAACCAAGCGCGGGTAAACGGCGGGAGTAACTATGACTCTCTTAAGGTAGCC
                *** ********************************************************

Fusk            AAATGCCTCGTCATCTAATTAGTGACGCGCATGAATGGATTAACGAGATTCCCACTGTCC
Penc            AAATGCCTCGTCATCTAATTAGTGACGCGCATGAATGGATTAACGAGATTCCCACTGTCC
Aspf            AAATGCCTCGTCATCTAATTAGTGACGCGCATGAATGGATTAACGAGATTCCCACTGTCC
Aspn            AAATGCCTCGTCATCTAATTAGTGACGCGCATGAATGGATTAACGAGATTCCCACTGTCC
Cana            AAATGCCTCGTCATCTAATTAGTGACGCGCATGAATGGATTAACGAGATTCCCACTGTCC
Cang            AAATGCCTCGTCATCTAATTAGTGACGCGCATGAATGGATTAACGAGATTCCCACTGTCC
Sacc            AAATGCCTCGTCATCTAATTAGTGACGCGCATGAATGGATTAACGAGATTCCCACTGTCC
Malg            AAATGCCTCGTCATCTAATTAGTGACGCGCATGAATGGATGAACGAGATTCCCACTGTCC
Cryn            AAATGCCTCGTCATCTAATTAGTGACGCGCATGAATGGATTAACGAGATTCCCACTGTCC
Cutd            AAATGCCTCGTCATCTAATTAGTGACGCGCATGAATGGATTAACGAGATTCCCACTGTCC
                **************************************** *******************

Fusk            CTATCTACTATCTAGCGAAACCACAGCCAAGGGAACGGGCTTGGCAGAATCAGCGGGGGA
Penc            CTATCTACTATCTAGCGAAACCACAGCCAGGGGAACGGGCCTGGCAGAATCAGC-GGGGA
Aspf            CTATCTACTATCTAGCGAAACCACAGCCAAGGGAACGGGCTTGGCAGAATCAGC-GGGGA
Aspn            CTATCTACTATCTAGCGAAACCACAGCCAAGGGAACGGGCTTGGCAGAATCAGC-GGGGA
Cana            CTATCTACTATCTAGCGAAACCACAGCCAAGGGAACGGGCTTGGCAGAATCAGC-GGGGA
Cang            CTATCTACTATCTAGCGAAACCACAGCCAAGGGAACGGGCTTGGCAGAATCAGC-GGGGA
Sacc            CTATCTACTATCTAGCGAAACCACAGCCAAGGGAACGGGCTTGGCAGAATCAGC-GGGGA
Malg            CTATCTACTATCTAGCGAAACCACAGCCAGGGGAACGGGCCTGGCAGAATCAGC-GGGGA
Cryn            CTATCTACTATCTAGCGAAACCACAGCCAAGGGAACGGGCTTGGCAGAATCAGC-GGGGA
Cutd            CTATCTACTATCTAGCGAAACCACAGCCAAGGGAACGGGCTTGGCAGAATCAGC-GGGGA
                ***************************** ********** ************* *****

Fusk            AAGAAGACCCTGTTGAGCTTGACTCTAGTTTGACATTGTGAAAAGACATAGGAGGTGTAG
Penc            AAGAAGACCCTGTTGAGCTTGACTCTAGTTTGACATTGTGAAAAGACATATGGGGTGTAG
Aspf            AAGAAGACCCTGTTGAGCTTGACTCTAGTTTGACATTGTGAAAAGACATATGGGGTGTAG
Aspn            AAGAAGACCCTGTTGAGCTTGACTCTAGTTTGACATTGTGAAAAGACATATGGGGTGTAG
Cana            AAGAAGACCCTGTTGAGCTTGACTCTAGTTTGACATTGTGAAAAGACATGGAGGGTGTAG
Cang            AAGAAGACCCTGTTGAGCTTGACTCTAGTTTGACATTGTGAAGAGACATAGAGGGTGTAG
Sacc            AAGAAGACCCTGTTGAGCTTGACTCTAGTTTGACATTGTGAAGAGACATAGAGGGTGTAG
Malg            AAGAAGACCCTGTTGAGCTTGACTCTAGTTTGACATTGTGAAGAGACATAGAGGGTGTAG
Cryn            AAGAAGACCCTGTTGAGCTTGACTCTAGTTTGACATTGTGAAAAGACATGGAGGGTGTAG
Cutd            AAGAAGACCCTGTTGAGCTTGACTCTAGTTTGACATTGTGAAAAGACATGGAGGGTGTAG
                ****************************************** ******    *******

Fusk            AATAGGTGGGAGCTTCGGCGCCGGTGAAATACCACTACTCCTATTGTTTTTTTACTTATT
Penc            AATAGGTGGGAGCTCCGGCGCCAGTGAAATACCACTACCTTTATCGTTTTTTTACTTATT
Aspf            AATAGGTGGGAGCTTCGGCGCCAGTGAAATACCACTACCTTTATCGTTTTTTTACTTATT
Aspn            AATAGGTGGGAGCTTCGGCGCCAGTGAAATACCACTACCTTTATCGTTTTTTTACTTATT
Cana            AATAAGTGGGAGCTTCGGCGCCGGTGAAATACCACTACCTCTATAGTTTTTTTACTTATT
Cang            CATAAGTGGGAGCTCCGGCGCCAGTGAAATACCACTACCTTTATAGTTTCTTTACTTATT
Sacc            AATAAGTGGGAGCTTCGGCGCCAGTGAAATACCACTACCTTTATAGTTTCTTTACTTATT
Malg            GATAAGTGGGAGCTCCGGCGCCAGTGAAATACCACTACCTTTATCGTCTTTTTACTTATT
Cryn            AATAAGTGGGAGCTTCGGCGCCGGTGAAATACCACTACCTCCATCGTTTTTTTACTTATT
Cutd            AATAAGTGGGAGCTTCGGCGCCGGTGAAATACCACTACCTCCATCGTTTTTTTACTTATT
                 *** ********* ******* ***************    ** ** * **********

Fusk            CAATGAAGCGGGGCTGGATTT--TCGTCCAACTTCTGGTTTTAAGG--TCCTTCGCGGGC
Penc            CAATGAAGCGGAACTGGGCTTCACCGCCCATCTTCTAGCGTTAAGG--TCCTTCGCGGGC
Aspf            CAATGAAGCGGAACTGGGCTTCACCGCCCATCTTCTGGCGTTAAGG--TCCTTCGCGGGC
Aspn            CAATGAAGCGGAACTGGGCTTCACCGCCCATCTTCTGGCGTTAAGG--TCCTTCGCGGGC
Cana            CAATGAAGCGGAGCTGGAGGTCAAACTCCACGTTCTAGCATTAAG---CCCTCT---GGG
Cang            CAATTAAGCGGAGCTGGAATTCATTTTCCACGTTCTAGCTTTCAAAGTGCCATTCGGTGC
Sacc            CAATGAAGCGGAGCTGGAATTCATTTTCCACGTTCTAGCATTCAAGGTCCCATTCGGGGC
Malg            CAATGAAGCGGAACTGGGCTTAACCGCCCACGTTTTGGCGTTAAGG--TCCTACGCGGGC
Cryn            CAATGAAGCGGAGCTGGGATGAAAGTCCCACCTTCTAGCGTTAAGG--TCGTTTACCGGC
Cutd            CAATGAAGCGGAACTGGGATTAACGTCCCACTTTTTGGCTTTAAGG--TCCTTTACGGGC
                **** ******  ****          ***  ** * *  ** *     *        *

Fusk            CGACCCGGGTTGAAGACATTGTCAGGTGGGGGAGTTTGGCTGGGGCGGCACATCTGTTAA
Penc            CGATCCGGGTTGAAGACATTGTCAGGT-GGGGAGTTTGGCTGGGGCGGCACATCTGTTAA
Aspf            CGATCCGGGTTGAAGACATTGTCAGGT-GGGGAGTTTGGCTGGGGCGGCACATCTGTTAA
Aspn            CGATCCGGGTTGAAGACATTGTCAGGT-GGGGAGTTTGGCTGGGGCGGCACATCTGTTAA
Cana            CGATCCGGGTTGAAGACATTGTCAGGT-GGGGAGTTTGGCTGGGGCGGCACATCTGTTAA
Cang            TGATCCGGGTTGAAGACATTGTCAGGT-GGGGAGTTTGGCTGGGGCGGCACATCTGTTAA
Sacc            TGATCCGGGTTGAAGACATTGTCAGGT-GGGGAGTTTGGCTGGGGCGGCACATCTGTTAA
Malg            TGATCCGGGTTGAAGACATTGTCAGGT-GGGGAGTTTGGCTGGGGCGGCACATCTGTTAA
Cryn            CGATCCGGGTTGAAGACATTGTCAGGT-GGGGAGTTTGGCTGGGGCGGCACATCTGTTAA
Cutd            TGATCCGGGTTGAAGACATTGTCAGGT-GGGGAGTTTGGCTGGGGCGGCACATCTGTTAA
```

```
                 ** *********************** ********************************

Fusk            ACCATAACGCAGGTGTCCTAAGGGGGTCTCATGGAGAACAGAAATCTCCAGTAGAGCAAA
Penc            ACAACAACGCAGGTGTCCTAAGGGGGACTCATGGAGAACAGAAATCTCCAGTAGAACAAA
Aspf            ACCACAACGCAGGTGTCCTAAGGGGGACTCATGGAGAACAGAAATCTCCAGTAGAACAAA
Aspn            ACCACAACGCAGGTGTCCTAAGGGGGACTCATGGAGAACAGAAATCTCCAGTAGAACAAA
Cana            ACGATAACGCAGGTGTCCTAAGGGGGACTCATGGAGAACAGAAATCTCCAGTAGAACAAA
Cang            ACGATAACGCAGATGTCCTAAGGGGGACTCATGGAGAACAGAAATCTCCAGTAGAACAAA
Sacc            ACGATAACGCAGATGTCCTAAGGGGGGCTCATGGAGAACAGAAATCTCCAGTAGAACAAA
Malg            ACAATAACGCAGGTGTCCTAAGGGGGACTCATGGAGAACAGAAATCTCCAGTAGAACAAA
Cryn            AAAATAACGCAGGTGTCCTAAGGGGGACTCATGGAGAACAGAAATCTCCAGTGGAACAAA
Cutd            AAGATAACGCAGGTGTCCTAAGGGGGACTCATGGAGAACAGAAATCTCCAGTGGAACAAA
                *  * ******* ************* ************************* ** ****

Fusk            AGGGCAAAAGTCCCCTTGATTTTGATTTTCAGTGTGAATACAAACCATGAAAGTGTGGCC
Penc            AGGGTAAAAGTCCCCTTGATTTTGATTTTCAGTGTGAATACAAACCATGAAAGTGTGGCC
Aspf            AGGGTAAAAGTCCCCTTGATTTTGATTTTCAGTGTGAATACAAACCATGAAAGTGTGGCC
Aspn            AGGGTAAAAGTCCCCTTGATTTTGATTTTCAGTGTGAATACAAACCATGAAAGTGTGGCC
Cana            AGGGTAAAAGTCCCCTTGATTTTGATTTTCAGTGTGAATACAAACCATGAAAGTGTGGCC
Cang            AGGGTAAAAGTCCCCTTGATTTTGATTTTCAGTGTGAATACAAACCATGAAAGTGTGGCC
Sacc            AGGGTAAAAGCCCCCTTGATTTTGATTTTCAGTGTGAATACAAACCATGAAAGTGTGGCC
Malg            AGGGTAAAAGTCCCCTTGATTTTGATTTTCAGTGTGAATACAAACCATGAAAGTGTGGCC
Cryn            AGGGTAAAAGTCCCCTTGATTTTGATTTTCAGTGTGAATACAAACCATGAAAGTGTGGCC
Cutd            AGGGTAAAAGTCCCCTTGATTTTGATTTTCAGTGTGAATACAAACCATGAAAGTGTGGCC
                **** ***** *************************************************

Fusk            TATCGATCCTTTAGTCCCTCGACATT-TGAGGCTAGAGGTGCCAGAAAAGTTACCACAGG
Penc            TATCGATCCTTTAGTCCCTCGAAATT-TGAGGCTAGAGGTGCCAGAAAAGTTACCACAGG
Aspf            TATCGATCCTTTAGTCCCTCGAAATT-TGAGGCTAGAGGTGCCAGAAAAGTTACCACAGG
Aspn            TATCGATCCTTTAGTCCCTCGAAATT-TGAGGCTAGAGGTGCCAGAAAAGTTACCACAGG
Cana            TATCGATCCTTTAGTCCCTCGGAATT-TGAGGCTAGAGGTGCCAGAAAAGTTACCACAGG
Cang            TATCGATCCTTTAGTCCCTCGGAATT-TGAGGCTAGAGGTGCCAGAAAAGTTACCACAGG
Sacc            TATCGATCCTTTAGTCCCTCGGAATT-TGAGGCTAGAGGTGCCAGAAAAGTTACCACAGG
Malg            TATCGAACCTTTAGTCCCTCGGAGTTATGAGGCTAGAGATGCCAGAAAAGTTACCACAGG
Cryn            TATCGATCCTTTAGTCCCTCGGAGTT-TGAGGCTAGAGGTGCCAGAAAAGTTACCACAGG
Cutd            TATCGATCCTTTAGTCCCTCGGAATT-TGAGGCTAGAGGTGCCAGAAAAGTTACCACAGG
                ****** **************   ** *********** *********************

Fusk            GATAACTGGCTTGTGGCGGCCAAGCGTTCATAGCGACGTCGCTTTTTGATCCTTCGATGT
Penc            GATAACTGGCTTGTGGCAGCCAAGCGTTCATAGCGACGTTGCTTTTTGATCCTTCGATGT
Aspf            GATAACTGGCTTGTGGCAGCCAAGCGTTCATAGCGACGTTGCTTTTTGATCCTTCGATGT
Aspn            GATAACTGGCTTGTGGCAGCCAAGCGTTCATAGCGACGTTGCTTTTTGATCCTTCGATGT
Cana            GATAACTGGCTTGTGGCAGTCAAGCGTTCATAGCGACATTGCTTTTTGATTCTTCGATGT
Cang            GATAACTGGCTTGTGGCAGTCAAGCGTTCATAGCGACATTGCTTTTTGATTCTTCGATGT
Sacc            GATAACTGGCTTGTGGCAGTCAAGCGTTCATAGCGACATTGCTTTTTGATTCTTCGATGT
Malg            GATAACTGGCTTGTGGCAGCCAAGCGTTCATAGCGACGTTGCTTTTTGATCCTTCGATGT
Cryn            GATAACTGGCTTGTGGCAGCCAAGCGTTCATAGCGACGTTGCTTTTTGATCCTTCGATGT
Cutd            GATAACTGGCTTGTGGCAGCCAAGCGTTCATAGCGACGTTGCTTTTTGATCCTTCGATGT
                ***************** * ***************** * ********** *********

Fusk            CGGTTTCTTCCTATCATACCGAAGCAGAATTCGGTAAGCGTTGGATTGTTCACCCACTAA
Penc            CGG-CTCTTCCTATCATACCGAAGCAGAATTCGGTAAGCGTTGGATTGTTCACCCACTAA
Aspf            CGG-CTCTTCCTATCATACCGAAGCAGAATTCGGTAAGCGTTGGATTGTTCACCCACTAA
Aspn            CGG-CTCTTCCTATCATACCGAAGCAGAATTCGGTAAGCGTTGGATTGTTCACCCACTAA
Cana            CGG-CTCTTCCTATCATACCGAAGCAGAATTCGGTAAGCGTTGGATTGTTCACCCACTAA
Cang            CGG-CTCTTCCTATCATACCGAAGCAGAATTCGGTAAGCGTTGGATTGTTCACCCACTAA
Sacc            CGG-CTCTTCCTATCATACCGAAGCAGAATTCGGTAAGCGTTGGATTGTTCACCCACTAA
Malg            CGG-CTCTTCCTATCATACCGAAGCAGAATTCGGTAAGCGTTGGATTGTTCACCCACTAA
Cryn            CGG-CTCTTCCTATCATACCGAAGCAGAATTCGGTAAGCGTTGGATTGTTCACCCACTAA
Cutd            CGG-CTCTTCCTATCATACCGAAGCAGAATTCGGTAAGCGTTGGATTGTTCACCCACTAA
                ***  *******************************************************

Fusk            TAGGGAACGTGAGCTGGGTTTAGACCGTCGTGAGACAGGTTAGTTTTACCCTACTGAT--
Penc            TAGGGAACGTGAGCTGGGTTTAGACCGTCGTGAGACAGGTTAGTTTTACCCTACTGAT-G
Aspf            TAGGGAACGTGAGCTGGGTTTAGACCGTCGTGAGACAGGTTAGTTTTACCCTACTGAT-G
Aspn            TAGGGAACGTGAGCTGGGTTTAGACCGTCGTGAGACAGGTTAGTTTTACCCTACTGAT-G
Cana            TAGGGAACGTGAGCTGGGTTTAGACCGTCGTGAGACAGGTTAGTTTTACCCTACTGAT-G
Cang            TAGGGAACGTGAGCTGGGTTTAGACCGTCGTGAGACAGGTTAGTTTTACCCTACTGAT-G
Sacc            TAGGGAACGTGAGCTGGGTTTAGACCGTCGTGAGACAGGTTAGTTTTACCCTACTGAT-G
Malg            TAGGGAACGTGAGCTGGGTTTAGACCGTCGTGAGACAGGTTAGTTTTACCCTACTGATGG
Cryn            TAGGGAACGTGAGCTGGGTTTAGACCGTCGTGAGACAGGTTAGTTTTACCCTACTGATGG
Cutd            TAGGGAACGTGAGCTGGGTTTAGACCGTCGTGAGACAGGTTAGTTTTACCCTACTGATGG
                **********************************************************

Fusk            GACCTCGCCGCAATGGTAATTCAGCTTAGTACGAGAGGAACCGCTGATTCAGATAATTGG
Penc            AAGGTCGCCGCAACGGTAATTCAATTTAGTACGAGAGGAACCGTTGATTCAGATAATTGG
Aspf            AAGGTCGCCGCAACGGTAATTCAATTTAGTACGAGAGGAACCGTTGATTCAGATAATTGG
Aspn            AAGGTCGCCGCAACGGTAATTCAATTTAGTACGAGAGGAACCGTTGATTCAGATAATTGG
Cana            AATGTTATCGCAATAGTAATTGAACTTAGTACGAGAGGAACCGTTCATTCAGATAATTGG
Cang            AATGTTACCGCAATAGTAATTGAACTTAGTACGAGAGGAACAGTTCATTCGGATAATTGG
Sacc            AATGTTACCGCAATAGTAATTGAACTTAGTACGAGAGGAACAGTTCATTCGGATAATTGG
Malg            AGGGTTATCGCAATAGTAATTCAACTTAGTACGAAAGGAACCGTTGATTCAGATAATTGG
Cryn            AGTGTCGTCGTAATAGTAATTGAGGGTAGTACGAGAGGAACTGCTCATTCGTATAATTGG
Cutd            AGGGTCATCGTAATAGTAATTGAGGGTAGTACGAGAGGAACTGCTCATTCGTATAATTGG
                    *   ** **  ****** *   ******** ****** * * ****  ********

Fusk            TTTTTGCGGCTGTCCGACCGGGCAGTGCCGCGAAGCTACCATCTGCTGGATAATGGCTGA
Penc            TTTTTGCGGCTGTCTGACCAGGCAGTGCCGCGACGCTACCATCTGCCGGATTATGGCTGA
Aspf            TTTTTGCGGCTGTCTGACCAGGCAGTGCCGCGACGCTACCATCTGCCGGATAATGGCTGA
Aspn            TTTTTGCGGCTGTCTGACCAGGCAGTGCCGCGACGCTACCATCTGCCGGATAATGGCTGA
Cana            TTTTTGCGGCTGTCTGATCAGGCAACGCCGCGAAGCTACCATCTGCTGGATTATGGCTGA
Cang            TTTTTGCGGCTGTCTGATCAGGCAATGCCGCGAAGCTACCATCCGCTGGATTATGGCTGA
Sacc            TTTTTGCGGCTGTCTGATCAGGCATTGCCGCGAAGCTACCATCCGCTGGATTATGGCTGA
Malg            TATTTGCATCTGTCCGATCGGGCAATGGTGCGAAGCTATCATCTGCTGGATAATGGCTGA
Cryn            TATTTGCGTCTGTCCGATCGGGCAATGACGCGAAGCTATCATACGCCAGATTATGGCTGA
Cutd            TATTTGCGGCTGTCCGATCGGGCAATGCCGCGAAGCTATCATACGCTAGATTATGGCTGA
                * *****  ***** ** * ****  *  **** **** ***  **  *** ********
               LR12 primer
Fusk            ACGCCTCTAAGTC
Penc            ACGCCTCTAAGTC
Aspf            ACGCCTCTAAGTC
Aspn            ACGCCTCTAAGTC
Cana            ACGCCTCTAAGTC
Cang            ACGCCTCTAAGTC
Sacc            ACGCCTCTAAGTC
Malg            ACGCCTCTAAGCC
Cryn            ACGCCTCTAAGTC
Cutd            ACGCCTCTAAGTC
                *********** *
```
