# Supplementary material for: Using nanopore sequencing to identify fungi from clinical samples with high phylogenetic resolution
Source: Sci Rep. 2023 Jun 16;13:9785. doi: 10.1038/s41598-023-37016-0 (PMC10275880; doi:10.1038/s41598-023-37016-0)
Supplement: Supplementary file 1 — Supplementary Information. [file 41598_2023_37016_MOESM1_ESM.zip › Supplementary_Information/Supplementary_Methods/Bioinformatics_pipelines.pdf]

Supplementary Methods

Bioinformatics pipelines

The following are operational examples of analyzing nanopore sequencing data (barcode01: RGN3 amplicons) from a 10-species mock-community sample. The commands (highlighted in a gray background) should be executed in the Terminal on a Mac, with line breaks ignored.

Directory structure

```
.
├── barcode01
│   ├── fastq_runid_###.fastq.gz
│   ├── fastq_runid_###.fastq.gz
│   ├── fastq_runid_###.fastq.gz
│   ├── ...
│   ├── ...
│   └── fastq_runid_###.fastq.gz
└── rpt.txt
```

The contents of "rpt.txt" are as follows:

```
aaaaaaaaaaaaaaaaaaaa
cccccccccccccccccccc
acacacacacacacacacac
agagagagagagagagagag
atatatatatatatatatat
cgcgcgcgcgcgcgcgcgcg
```

Merge compressed FASTQ files (.gz format files) into a single file.

```
cat barcode01/*.fastq.gz > 01_bc01.fastq.gz
```

Extract the compressed file.

```
gunzip 01_bc01.fastq.gz
```

Filter reads by length (1420–1880 bases) and average quality score (Q15) using SeqKit.

```
seqkit seq -m 1420 -M 1880 -Q 15 01_bc01.fastq > 02_bc01.fastq
```

Remove reads with low-complexity sequences.

```
seqkit grep -vsif rpt.txt 02_bc01.fastq > 03_bc01.fastq
```

Create an output directory and run VSEARCH to cluster reads at 88% sequence similarity.

```
mkdir 04_bc01 | vsearch --cluster_fast 03_bc01.fastq --id 0.88 --strand
both --clusters 04_bc01/clus
```

Select output files (10 clusters) and rename them with **consecutive numbers** (filename: 05_cluster_**01**.fasta–05_cluster_**10**.fasta).

Split cluster files into two parts and extract centroid sequences.

```
for f in 05*; do seqkit split -p 2 $f; done

for x in `seq -f '%02g' 1 10`; do seqkit range -r -1:-1 05_cluster_${x}.fasta.split/05_cluster_${x}.part_001.fasta -w 0 > 06_centroid_${x}.fasta; done
```

Randomly subsample 300 reads.

```
for f in 05*.fasta; do seqkit shuffle -s 23 $f | seqkit head -n 300 -w 0 > ${f/05_cluster/07_subread}; done
```

Activate the environment and run Medaka to generate consensus sequences.

```
source ~/medaka-1.6.1/venv/bin/activate

for x in `seq -f '%02g' 1 10`; do medaka_consensus -d 06_centroid_${x}.fasta -i 07_subread_${x}.fasta -o 08_medaka_${x} -m r941_min_hac_g507; done

deactivate
```

Copy and rename consensus sequence files.

```
for x in `seq -f '%02g' 1 10`; do cp 08_medaka_${x}/consensus.fasta 09_consensus_${x}.fasta; done
```

Trim primer sequences manually to create query sequences for the BLAST search (filename: query_01.fasta–query_10.fasta).

Create a "database" directory where all the relevant database files are stored.
Download genome assemblies (named "genomes.fna") and build a BLAST database (title: Fungi_ref).

```
makeblastdb -in genomes.fna -out Fungi_ref -dbtype nucl -hash_index -parse_seqids
```

Run BLAST+ for taxonomic assignment.
#default pairwise format (fmt0)

```
for f in query*.fasta; do blastn -query $f -db database/Fungi_ref -out ${f/.fasta/_fmt0.txt} -num_threads 4; done
```

#tabular view format (fmt6)

```
for f in query*.fasta; do blastn -query $f -db database/Fungi_ref -out ${f/.fasta/_fmt6.txt} -outfmt "6 qaccver saccver qcovs bitscore nident mismatch gaps pident evalue" -num_threads 4; done
```
